# Better-than-$\frac{4}{3}$-approximations for leaf-to-leaf tree and connectivity augmentation

**DOI:** 10.1007/s10107-023-02018-3

**Published:** 2023-09-26

**Authors:** Federica Cecchetto, Vera Traub, Rico Zenklusen

**Affiliations:** 1 Department of Mathematics, ETH Zurich, Zurich, Switzerland; 2 Research Institute for Discrete Mathematics and Hausdorff Center for Mathematics, University of Bonn, Bonn, Germany

**Keywords:** Approximation algorithms, Connectivity augmentation, Combinatorial optimization, Tree augmentation, 90C27, 05C85, 68W25

## Abstract

The Connectivity Augmentation Problem (CAP) together with a well-known special case thereof known as the Tree Augmentation Problem (TAP) are among the most basic Network Design problems. There has been a surge of interest recently to find approximation algorithms with guarantees below 2 for both TAP and CAP, culminating in the currently best approximation factor for both problems of 1.393 through quite sophisticated techniques. We present a new and arguably simple matching-based method for the well-known special case of leaf-to-leaf instances. Combining our work with prior techniques, we readily obtain a $(4/3+\varepsilon )$-approximation for Leaf-to-Leaf CAP by returning the better of our solution and one of an existing method. Prior to our work, a $4/3$-guarantee was only known for Leaf-to-Leaf TAP instances on trees of height 2. Moreover, when combining our technique with a recently introduced stack analysis approach, which is part of the above-mentioned 1.393-approximation, we can further improve the approximation factor to 1.29, obtaining for the first time a factor below $\frac{4}{3}$ for a nontrivial class of TAP/CAP instances.

## Introduction

The Connectivity Augmentation Problem (CAP) is one of the most elementary Network Design problems. It asks to increase the edge-connectivity of a graph by one unit in the most economical way by adding edges/links from a given set. Formally, one is given a graph $G=(V,E)$ and an additional link set , and the task is to determine a smallest size set of links $U\subseteq L$ such that the edge-connectivity of $(V,E\cup U)$ is strictly larger than that of *G*. A famous special case of CAP is the Tree Augmentation Problem (TAP), where the given graph *G* is a spanning tree. Already TAP is well-known to be APX-hard (see [21], which presents an extension of a construction used in [13] to prove NP-hardness), even on trees of diameter 5 with all links going between pairs of leaves, i.e., *leaf-to-leaf instances*. This motivated the search for strong constant-factor approximations. There has been extensive work during the last decades on the approximability of both TAP and CAP, and also their weighted counterparts where each link has a cost and instead of minimizing the size of *U* the goal is to minimize its cost.

For CAP (and therefore also for TAP), multiple 2-approximations have been known for a long time, including through classical techniques like primal-dual algorithms and iterative rounding (see [13, 15, 19, 20]). It was an important stepping stone to reach algorithms with an approximation guarantee below 2. During the last decades, a long line of research led to multiple approaches that beat the approximation factor 2 for TAP and CAP through the introduction of a rich set of techniques [1, 3–5, 7, 8, 10, 11, 13, 16, 16, 20, 22, 23, 25, 26]. This led to the current state-of-the art approximation factor of 1.393 [3], which is currently the best one for both TAP and CAP. The factor of 1.393 was obtained through a combination of quite sophisticated techniques, and the obtained factor is neither natural nor is it likely to be the “right” answer, i.e., it seems very likely that approximation algorithms with better approximation guarantees should exist.

A natural question we are interested in, is whether there may be a clean algorithm leading to a $4/3$-approximation. This factor appears in other, related Network Design problems, in particular in the (unweighted) 2-Edge-Connected Spanning Subgraph problem (2-ECSS). In 2-ECSS, one starts with an empty graph *G* and the task is to pick a smallest number of links to obtain a 2-edge-connected graph spanning all vertices. Hence, instead of starting from a spanning tree to obtain a 2-edge-connected graph as in TAP, one starts with an empty graph. Recent advances on 2-ECSS led to the best-known approximation factor of 1.326 [14]. In the context of TAP and CAP, we still lack appropriate techniques to reach such factors, and progress along this line has only been achieved for quite restricted special cases. More precisely, for TAP, a factor of $4/3$ is known to be achievable if we are given an optimal solution to the natural LP relaxation, known as the *cut-LP*, that has the additional property of being half-integral [6] or, more generally, fulfills that each non-zero entry is at least $1/2$ [18]. However, the *cut-LP* is in general not a half-integral LP [6] and it may not contain any optimal point where each non-zero is at least $1/2$. Moreover, an approach for TAP was presented in [24] that leads to a $4/3$-approximation for Leaf-to-Leaf TAP instances on trees of height at most 2. Finally, for CAP, we are unaware of any nontrivial class of instances where such factors, or even factors below the currently best 1.393-approximation, are known. Even for TAP, we are not aware of a natural special case for which ratio below $4/3$ is known.

The goal of this paper is to make first progress in this regard for the case of Leaf-to-Leaf CAP (and therefore also TAP) instances. (We formally define Leaf-to-Leaf CAP instances in Sect. 1.1 and show their relation to Leaf-to-Leaf TAP and also Leaf-to-Leaf Cactus Augmentation, which is a well-known connection on which we heavily rely later on.) We think of Leaf-to-Leaf CAP instances as an appealing class because of the following reasons. First, they comprise a large family of nontrivial TAP/CAP instances, which have already been studied both in the context of TAP [24] and CAP [27]. Second, the best known hardness results for TAP/CAP are based on leaf-to-leaf instances.

We also note that, for the weighted settings of TAP/CAP, any instance can be reduced in an approximation-preserving way to a weighted leaf-to-leaf one; hence, the difference between leaf-to-leaf and general instances vanishes in the weighted settings.^1^

### Preliminaries

Consider a CAP instance $(G=(V,E),L)$ on a graph *G* that is *k*-edge-connected (but not $(k+1)$-edge-connected). Hence, the goal is to add a smallest number of links $U\subseteq L$ such that $(V,E\cup U)$ is $(k+1)$-edge-connected. By Menger’s Theorem, for a set $U\subseteq L$, the graph $(V,E\cup U)$ is $(k+1)$-edge-connected if and only if each minimum cut in *G* is covered by at least one link of *U*. (By a *cut* *C*, we refer to a vertex set $C \subseteq V$, and an edge/link is said to *cover* *C* if it has precisely one of its endpoints in *C*.) (*G*, *L*) is a *Leaf-to-Leaf CAP* instance if, for each link $\{u,v\}\in L$, both *u* and *v* are contained in an inclusion-wise minimal minimum cut. The *inclusion-wise minimal minimum cuts* in *G* are minimum cuts $A\subseteq V$ for which no other minimum cut $B \subseteq V$ satisfies $B\subseteq A$. Classic uncrossing results imply that the inclusion-wise minimal minimum cuts of a graph form a family of disjoint sets. Note that in case of a tree, these cuts are singleton cuts only containing a single leaf vertex. Thus, a leaf-to-leaf instance for a tree indeed maps to only having links with both endpoints being leaves.

**Fig. 1:**
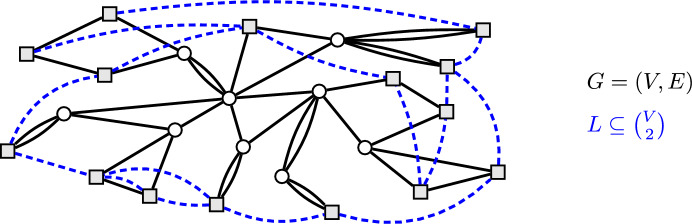
The black solid graph $G=(V,E)$ is a cactus. Its vertices of degree 2 are called *leaves* or *terminals* and are depicted as gray squares. Together with the dashed edges, which represent the links *L* and only go between leaves, we obtain a Leaf-to-Leaf CacAP instance

For CAP, it is often significantly more convenient to first use a well-known reduction to the *Cactus Augmentation Problem* (CacAP), which is the special case of CAP where the underlying graph is a cactus, i.e., it is a connected graph with each edge being contained in a unique cycle. See Fig. 1 for an example.

This reduction from CAP to CacAP is approximation preserving and an immediate consequence of the fact that the minimum cuts in a graph can be represented by a cactus (see [9]). Vertices of degree 2 in a cactus are also called *terminals* or *leaves* and the reduction from CAP to CacAP implies that Leaf-to-Leaf CAP instances are transformed into Leaf-to-Leaf CacAP instances. (See Fig. 1 for an example of a Leaf-to-Leaf CacAP instance.) Due to this equivalence, we therefore focus on the more structured Leaf-to-Leaf CacAP instances.

Note that any Leaf-to-Leaf TAP instance $(G=(V,E),L)$ can easily be cast as a Leaf-to-Leaf CacAP instance by adding for each edge $e\in E$ a parallel edge.

### Our results

Our main result is the following.

#### Theorem 1

There is a 1.29-approximation algorithm for Leaf-to-Leaf CAP (and therefore also Leaf-to-Leaf TAP).

In the context of leaf-to-leaf instances, this improves on the 1.393-approximation of [3] (which also works for CAP instances that are not leaf-to-leaf) and also improves on (and is applicable to a much broader set of instances than) the $\frac{4}{3}$-approximation for Leaf-to-Leaf TAP on trees of height 2 of [24].

Our main technical contribution, which is the central ingredient of our approach, is an arguably elegant technique to find a good CAP solution by first computing a maximum weight matching over the links with respect to judiciously chosen weights. This matching is then complemented through a simple LP to an actual CAP solution. We provide a detailed discussion of this matching-based approach in Sect. 2.

We highlight that [24], for the special case of TAP, also used an approach based on first computing a matching and extending it to a solution. Their approach to extend the matching to a solution uses a credit-based argument, whereas our matching-based approach relies on an LP. An LP-based approach also allows us to leverage recently introduced techniques from [1, 3, 11, 16].

### Brief overview of main components

**Fig. 2:**
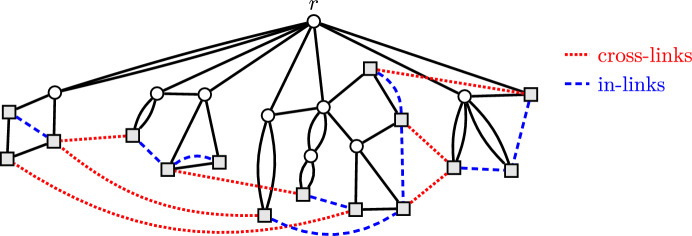
A Leaf-to-Leaf CacAP instance with root *r* on top. The leaves are drawn as gray squares. The dashed lines represent the links, which can be partitioned into cross-links (dotted in red) and in-links (dashed in blue)

Similar to prior approaches in the field, we provide a guarantee that can be expressed in terms of different link types. To this end, given a CacAP instance $(G=(V,E),L)$, we can fix an arbitrary root $r\in V$ and define link types with respect to this root as follows. A link $\{u,v\}\in L$ is called *cross-link*, if its two endpoints *u* and *v* lie in different connected components of $G-r$, where $G-r :=G[V\setminus \{r\}]$ is the subgraph of *G* induced by $V\setminus \{r\}$, i.e., the graph obtained from *G* by removing the root and all edges incident with it. All other links are called *in-links*. (See Fig. 2.) We denote the sets of all in-links and cross-links by $L_{\textrm{in}}$ and $L_{\textrm{cross}}$, respectively, and for any link set $U\subseteq L$, we use the shorthands $U_{\textrm{in}}:=U\cap L_{\textrm{in}}$ and $U_{\textrm{cross}}:=U \cap L_{\textrm{cross}}$.

We give a procedure that returns a solution with the guarantee stated below.

#### Theorem 2

For any Leaf-to-Leaf CacAP instance (*G*, *L*), we can in polynomial time compute a solution $F \subseteq L$ such that $|F| \le |H| + \frac{1}{2}|H_{\textrm{in}}|$ for any solution *H* of the instance.

In particular, the cardinality of the solution *F* we return is no larger than the cardinality of any optimal solution *H* plus half of the number of in-links of *H*. Clearly, this immediately implies a $3/2$-approximation for Leaf-to-Leaf CacAP (and therefore also for Leaf-to-Leaf CAP). The guarantees obtained through Theorem 2 are a strengthening, for the leaf-to-leaf case, of guarantees obtainable by prior methods, in particular one developed in [11] for TAP and extended in [3] to CacAP, which leads to a guarantee of $|F|\le |H| + |H_{\textrm{in}}|$.

Our approach readily leads to better factors than $3/2$ when combined with prior techniques that have been developed recently and can be translated to the leaf-to-leaf setting. These approaches are based on a reduction introduced in [1], and later extended in [3, 16], which first reduces the given instance to a better structured one, known as *k*-wide (for constant *k*).

#### Definition 3

(*k*-wide CacAP) Let $k\in \mathbb {Z}_{\ge 1}$. A CacAP instance (*G*, *L*) is *k*-*wide* if there is a vertex *r* such that each connected component of $G-r$ contains at most *k* leaves of *G*. We call *r* a *k*-*wide root* of *G*, or simply a *root*. Moreover, for each vertex set $W\subseteq V\setminus \{r\}$ of a connected component of $G-r$, we call $G[W\cup \{r\}]$ an *r*-principal subcactus of *G*.

In particular, the Leaf-to-Leaf CacAP instance highlighted in Fig. 2 is 6-wide, with respect to the indicated root *r*, and has 4 principal subcacti.

The following statement shows that, to obtain approximation algorithms for Leaf-to-Leaf CacAP, it suffices to consider *O*(1)-wide instances (up to an arbitrarily small error in the approximation factor).

#### Theorem 4

Let $\alpha \ge 1$, $\varepsilon > 0$, and $k:=\frac{256(16+3\varepsilon )}{\varepsilon ^3}$. Given an $\alpha$-approximation algorithm $\mathcal {A}$ for *k*-wide Leaf-to-Leaf CacAP instances, there is an $\alpha \cdot (1+\varepsilon )$-approximation algorithm $\mathcal {B}$ for (unrestricted) Leaf-to-Leaf CacAP that calls $\mathcal {A}$ at most polynomially many times and performs further operations taking polynomial time.

Theorem 4 (with a slightly different constant *k*) has been proven for (non leaf-to-leaf) CacAP [3]. As we discuss later, a slight modification of the reduction used in [3] allows for translating this result to the leaf-to-leaf setting, where we want to make sure that the *O*(1)-wide CacAP instance maintains the property of being also leaf-to-leaf if the original instance was leaf-to-leaf.

On *O*(1)-wide CacAP instances (even weighted ones), we can leverage the following result from [3], which is a consequence of a technique in [2].

#### Lemma 5

([3]) For any weighted *k*-wide CacAP instance $(G=(V,E),L)$ with link costs $c\in \mathbb {R}_{\ge 0}^L$, we can compute in time $3^k \texttt {poly}(|V|)$ a CacAP solution $F\subseteq L$ with $c(F) \le c(H) + c(H_{\textrm{cross}})$ for any solution *H* of the instance.

The above statement is obtained by solving independently and optimally the CacAP problems on each principal subcactus of a given *k*-wide instance and then returning the union of all these solutions. This is the step that requires *k* to be constant to be executable in polynomial time.

Note how the guarantee given by Lemma 5 is complementary to the one we obtain through the matching-based procedure described in Theorem 2. By returning the better of the two, we immediately obtain a $4/3$-approximation for *O*(1)-wide Leaf-to-Leaf CacAP.

#### Corollary 6

Let $(G=(V,E),L)$ be an *O*(1)-wide Leaf-to-Leaf CacAP instance. Computing a solution $F_1\subseteq L$ as claimed by Theorem 2 and a solution $F_2\subseteq L$ as claimed by Lemma 5 (with *c* being unit weights), and returning the one of smaller cardinality, leads to a $4/3$-approximation.

#### Proof

Let $\textrm{OPT}\subseteq L$ be an optimal solution of (*G*, *L*). By Theorem 2, we have $|F_1| \le |\textrm{OPT}| + \frac{1}{2}|\textrm{OPT}_{\textrm{in}}|$, and Lemma 5 provides $|F_2|\le |\textrm{OPT}| + |\textrm{OPT}_{\textrm{cross}}|$. Hence, the better of the two has size

$$\begin{aligned} \min \{|F_1|,|F_2|\} \le \frac{2}{3} |F_1| + \frac{1}{3} |F_2| \le |\textrm{OPT}| + \frac{1}{3} |\textrm{OPT}_{\textrm{in}}| + \frac{1}{3} |\textrm{OPT}_{\textrm{cross}}| = \frac{4}{3}|\textrm{OPT}|, \end{aligned}$$

as desired. $$\square $$

Thus, Corollary 6 immediately implies, together with Theorem 4, that there is a $(4/3+\varepsilon )$-approximation for Leaf-to-Leaf CacAP. Finally, a recently introduced technique based on stack analysis [3] allows for obtaining approximation factors below $4/3$, and implies the claimed 1.29-approximation for Leaf-to-Leaf CacAP (and therefore also Leaf-to-Leaf CAP) as stated in Theorem 1.

### Organization of paper

In Sect. 2, we introduce our main new technical ingredient, namely a simple matching-based approach to derive Leaf-to-Leaf CacAP (or TAP) solutions, which leads to Theorem 2. The reduction to *O*(1)-wide Leaf-to-Leaf CacAP instances is discussed in Sect. 3, with some additional explanation of how precisely we reuse results from prior work in Appendix A. Sect. 4 discusses how the stack analysis approach of [3] allows for obtaining approximation factors below $4/3$-factor, leading to the claimed 1.29-approximation for Leaf-to-Leaf CAP. Appendix B contains some further details on the stack analysis presented in [3].

## A matching-based approach

We now describe a new matching-based approach, which leads to Theorem 2. In fact, we will show a slight generalization of Theorem 2, which applies to a slightly larger problem class which we call *leaf-to-leaf+ instances*. This will be useful later on when we combine this matching-based approach with other algorithms to obtain our main result, Theorem 1.

### Definition 7

(Leaf-to-Leaf+ CacAP instance) A CacAP instance $(G=(V,E),L)$ is a *leaf-to-leaf+ instance* if it has a root $r\in V$ such that every endpoint of a link in *L* is the root or a leaf of *G*.

The main result of this section is the following, which immediately implies Theorem 2.

### Theorem 8

For any Leaf-to-Leaf+ CacAP instance (*G*, *L*), we can in polynomial time compute a solution $F\subseteq L$ such that $|F|\le |H| + \frac{1}{2}|H_{\textrm{in}}|$ for any solution *H* of the instance.

To describe this matching-based approach, consider a Leaf-to-Leaf+ CacAP instance $\mathcal {I}=(G=(V,E),L)$ together with a root $r\in V$. We denote by $\mathcal {C}$ the set of 2-cuts of *G*, where, by convention, we only consider cuts not containing *r*, i.e.,

$$\begin{aligned} \mathcal {C} :=\left\{ C\subseteq V\setminus \{r\} :|\delta _E(C)| = 2\right\} . \end{aligned}$$

Hence, the task is to find a smallest link set covering all sets in $\mathcal {C}$.

**Fig. 3:**
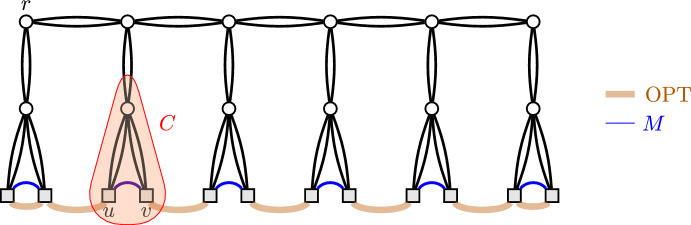
A Leaf-to-Leaf TAP instance, represented as a CacAP instance. The links are shown as blue lines and thick orange lines. The thick orange links show an optimal solution, which has cardinality 7. The blue are the unique maximum cardinality matching *M* on the link set. However, complementing the matching *M* to a solution requires at least 5 extra links (all orange/$\textrm{OPT}$ ones except for the left-most and right-most one), leading to a solution of cardinality at least 12. By making the example wider, the ratio between the solution obtained by (optimally) complementing *M* and $\textrm{OPT}$ approaches 2. The blue link $\{u,v\}$ is an example of a bad link, because there is a 2-cut *C*, highlighted in red, such that each link covering *C* has either *u* or *v* as one of its endpoints

We will first compute a large matching on the leaves *T* of *G* and then show that we can cheaply complete the matching to a solution *F* with the desired properties. On a high level, it seems intuitive that good solutions contain large matchings. In particular, a simple lower bound on the number of links needed in any solution is given by $|T|/2$, because each leaf needs to have a link incident with it. Any instance with an optimal solution close to this lower bound must thus contain a very large matching. However, simply computing a maximum cardinality matching and then completing it to a solution does not generally lead to strong solutions. See Fig. 3 for an example.

As we formalize in the following, a key reason for a matching not to have a good completion is that it contains a certain type of links, which we call *bad*. For the special case of TAP, Maduel and Nutov [24] already identified these links as being undesirable and called them *redundant*. A related notion used in the field is the one of *twin links*, which can be seen to be a special case of what we call bad links in the context of leaf-to-leaf instances. (See Kortsarz and Nutov [22] for a formal definition of twin links.)

### Definition 9

(Bad link) A link $\{u,v\}\in L$ is *bad* if there is a cut $C\in \mathcal {C}$ with $u,v\in C$ and such that any link covering *C* is incident to one of *u* and *v*. In this case, we say that $\{u,v\}$ is *bad with respect to* *C*.

Fig. 3 highlights an example of a bad link.

The key lemma is the following, which we show in Sect. 2.1. In words, it says that large matchings without bad links can be cheaply augmented to a CacAP solution.

### Lemma 10

Given a feasible Leaf-to-Leaf+ CacAP instance (*G*, *L*) and a matching $M\subseteq L$ on the leaves *T* of *G* without bad links, we can in polynomial time find a CacAP solution $F\subseteq L$ with $M\subseteq F$ such that:

1 $$\begin{aligned} |F|\le |M|+\frac{1}{2}|M_{\textrm{in}}|+(|T| - 2|M|). \end{aligned}$$

Algorithm 1 now simply computes a matching without bad links that minimizes the right-hand side of (1), and completes it to a solution with the guarantee claimed by Lemma 10. Note that finding a matching without bad links that minimizes

$$\begin{aligned} |M|+\frac{1}{2}|M_{\textrm{in}}| + (|T| - 2|M|) = -|M_{\textrm{cross}}| - \frac{1}{2}|M_{\textrm{in}}| + |T|, \end{aligned}$$

can easily be done by deleting all bad links and non leaf-to-leaf links and finding a maximum weight matching over the remaining links where each cross-link has a weight of 1 and each in-link has a weight of $1/2$. 
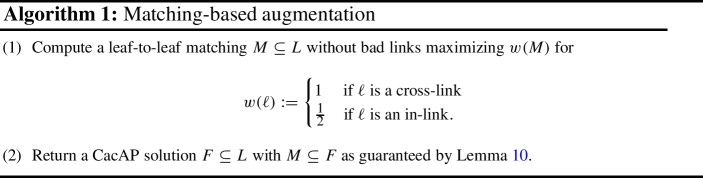


To derive that Algorithm 1 leads to a solution with the guarantees claimed by Theorem 8, we show that there exist matchings without bad links that lead to a cheap CacAP solution through Lemma 10.

### Lemma 11

For any Leaf-to-Leaf+ CacAP instance (*G*, *L*), there exists a matching $M\subseteq L$ on the leaves of *G* without bad links such that

$$\begin{aligned} |M|+\frac{1}{2}|M_{\textrm{in}}|+(|T| - 2|M|) \le |H| + \frac{1}{2}|H_{\textrm{in}}| \end{aligned}$$

for any solution *H* of the instance.

Finally, Theorem 8 is a straightforward consequence of the above statements, which imply that Algorithm 1 returns a CacAP solution with the desired guarantees.

### Proof of Theorem 8

As discussed, in the first step, Algorithm 1 finds in polynomial time a matching *M* that minimizes the right-hand side of (1). Then, Lemma 11 implies that the solution obtained from this matching *M* through Lemma 10, which is the one returned in Algorithm 1, has the desired guarantees. $\square$

In the rest of this section, we provide proofs for Lemma 10 and Lemma 11 in Sect. 2.1 and 2.2, respectively.

### Completing matchings to CacAP solutions (proof of Lemma 10)

In this section we provide details on the steps performed in our algorithm to obtain a solution $F\supseteq M$ from a leaf-to-leaf matching *M* without bad links as claimed in Lemma 10. To extend *M* to a CacAP solution $M\cup U$, for some link set $U\subseteq L$, we need that *U* covers all 2-cuts not yet covered by *M*. Hence, $M\cup U$ is a CacAP solution if and only if $U\cap \delta _L(C)\ne \emptyset$ for all $C\in \mathcal {C}^M$, where

$$\begin{aligned} \mathcal {C}^M :=\bigl \{ C \subseteq V\setminus \{r\} \, \ |\delta _E(C)|=2 \text { and } \delta _L(C)\cap M=\emptyset \bigr \}. \end{aligned}$$

To find a good extension *U*, we use the bi-directed LP that is integral, which is an idea introduced in [3]. More precisely, we use the following directed link set, which contains for each original link two antiparallel directed links:

$$\begin{aligned} \vec {L}:=\bigcup _{\{u,v\}\in L}\left\{ (u,v),(v,u)\right\} , \end{aligned}$$

and solve the following LP to find a good extension *U*:

dir-LP $$\begin{aligned} \begin{array}{crclc} \min &{} x(\vec {L}) &{} &{} &{} \\ &{} x(\delta ^-_{\vec {L}}(C))\ {} &{}\ge &{}1 &{} \forall \; C\in \mathcal {C}^M \\ &{} x &{} \in &{}\mathbb {R}^{\vec {L}}_{\ge 0}. &{} \end{array} \end{aligned}$$

In words, the LP requires one to “buy” directed links, and a cut is only counted as covered if a directed link is entering it.^2^ Whereas (dir-LP) is thus not a relaxation of the problem of finding the best completion *U* for *M*, it can be shown to be integral with extreme points being $\{0,1\}$-vectors (see Lemma 13). Moreover, it has an optimal objective value that is no more than twice as expensive as the optimal completion. This follows from the observation that one can set, for each link $\{u,v\}$ in an optimal completion $U^*$, the values of *x* for both (*u*, *v*) and (*v*, *u*) to 1 to obtain a feasible solution for (dir-LP).

In order to obtain a solution *F* with $M\subseteq F$, we compute an optimal extreme point solution $x^*$ of (dir-LP), which is a $\{0,1\}$-vector (see Lemma 13). Then we can define

$$\begin{aligned} U&:=\{\{u,v\}\in L :x^*((u,v))=1 \text { or } x^*((v,u))=1\} \end{aligned}$$

and set

$$\begin{aligned} F&:=U\cup M. \end{aligned}$$

We will show that *F* fulfills the guarantees claimed by Lemma 10.

First, we observe that $x^*$ is indeed a $\{0,1\}$-vector. We recall the definition of *residual instance* from [3, Definition 17], which will be useful when considering instances in which some links have been contracted.

#### Definition 12

(residual instance) Let $\mathcal {I}=(G,L)$ be a CacAP instance and let $L'\subseteq L$. Let $L'=\{\ell _1, \ldots , \ell _h\}$ be a numbering (ordering) of the links in $L'$. The *residual instance* of $\mathcal {I}$ with respect to $L'$ and this numbering is the instance that arises by performing the following contraction operation sequentially for each link $\ell =\ell _1$ up to $\ell =\ell _h$: contract all vertices that are on every *u*-*v* path in the cactus, where *u* and *v* are the endpoints of $\ell$, into a single vertex.

It is known that any contraction order leads to the same outcome (c.f. Lemma 18 in [3]) and hence we will in the following simply talk about the residual instance of $\mathcal {I}$ with respect to $L'$ without specifying an order of the links in $L'$. Moreover, a residual instance with respect to some link set $L'$ is a CacAP instance whose 2-cuts correspond precisely to the 2-cuts in *G* that have not been covered by $L'$ (Lemma 19 in [3]). This implies that a link set *F* is a feasible solution for the residual instance of $\mathcal {I}$ with respect to $L'$ if and only if $F\cup L'$ is a feasible solution for the instance $\mathcal {I}$ (Corollary 20 in [3]). The following statement follows by prior work on the integrality of covering cuts of an intersecting family (see [12] and discussion therein). For completeness, we provide a short proof below.

#### Lemma 13

Each extreme point of the feasible region of (dir-LP) is contained in $\{0,1\}^{\vec {L}}$.

#### Proof

We consider the residual instance $(G^M, L^M)$ with respect to the matching *M*. Then, as proved in [3, Lemma 19], one can observe that the 2-cuts $\mathcal {C}_{G^M}$ of the residual instance correspond precisely to the cuts in $\mathcal {C}^M$. By Lemma 14 in [3], the LP

$$\begin{aligned} \min \left\{ x(\vec {L}): x \in \mathbb {R}^{\vec {L}}_{\ge 0},\ x\left( \delta ^-_{\vec {L}}(C)\right) \ge 1 \text { for all }C\in \mathcal {C}_{G^M} \right\} \end{aligned}$$

is integral and hence also (dir-LP) is integral. Hence, all that remains to be shown is that the coordinates of extreme points are not just integral but each one of them is either zero or one. Observe first, that (dir-LP) contains non-negativity constraints, thus each coordinate of any extreme point is non-negative. Finally, we show that any point $x\in \mathbb {R}^{\vec {L}}_{\ge 0}$ feasible for (dir-LP) with $x(\ell )>1$ for some $\ell \in \vec {L}$ cannot be an extreme point. Indeed, for such a point *x*, both increasing or decreasing $x(\ell )$ by a small quantity will lead to other feasible points in (dir-LP), which contradicts the assumption of *x* being an extreme point.

$$\square $$

To show that the completion *U* computed through obtaining an extreme point solution to (dir-LP) satisfies $|U|\le \frac{1}{2}|M_{\textrm{in}}| + (|T|- 2|M|)$, it remains to show that the optimal value of (dir-LP) is at most $\frac{1}{2}|M_{\textrm{in}}| + (|T|-2|M|)$. We will prove this in two steps. First, we observe that the statement holds if we only look at a subset of constraints consisting of a laminar subfamily $\mathcal {L}\subseteq \mathcal {C}^M$, where we recall that $\mathcal {C}^M \subseteq \mathcal {C}$ are all 2-cuts that are not covered by *M*. Note that this case includes Leaf-to-Leaf+ TAP, where the set of all minimum cuts form a laminar family. In a second step, we leverage this result on laminar cuts to obtain the desired upper bound on (dir-LP) also for Leaf-to-Leaf+ CacAP instances, as desired.

#### Lemma 14

Let $\mathcal {L}\subseteq \mathcal {C}^M$ be a laminar family. Then the optimum value of the LP

2 $$\begin{aligned} \min \Bigl \{ x(\vec {L}): x\in \mathbb {R}^{\vec {L}}_{\ge 0}, x\bigl (\delta ^-_{\vec {L}}(C)\bigr ) \ge 1 \text { for all }C\in \mathcal {L}\Bigr \} \end{aligned}$$

is at most $\frac{1}{2}|M_{\textrm{in}}| + (|T| -2 |M|)$.

#### Proof

**Fig. 4:**
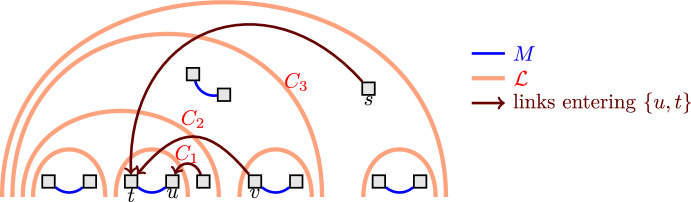
The link (*s*, *t*) is the maximal link entering *t* because $\mathcal {C}^{(s,t)}=\{C_1,C_2,C_3\} \supseteq \{C_1,C_2\}=\mathcal {C}^{(v,t)}$. Similarly, the link (*s*, *t*) is also the maximal link entering $\{t,u\}$

We construct a feasible solution $x^M$ for (2) with $x^M(L) \le \frac{1}{2}|M_{\textrm{in}}| + (|T| -2 |M|)$. For a link $\ell \in \vec {L}$, let

$$\begin{aligned} \mathcal {C}^{\ell } :=\{ C\in \mathcal {L}: \ell \in \delta ^-_{\vec {L}}(C) \} \end{aligned}$$

be the set of cuts in $\mathcal {L}$ that are covered by $\ell$. For a terminal $t\in T$, we call a link $(s,t)\in \vec {L}$ a *maximal link entering* *t* if the set $\mathcal {C}^{(s,t)}$ is inclusion-wise maximal among all links entering *t*, i.e., no other link $(\bar{s},t)$ entering *t* satisfies $\mathcal {C}^{(s,t)} \subsetneq \mathcal {C}^{(\bar{s},t)}$. Similarly, for a link $\{v,w\}\in M$, we call $\ell \in \delta ^-_{\vec {L}}(\{v,w\})$ a *maximal link entering* $\{v,w\}$ if the set $\mathcal {C}^{\ell }$ is inclusion-wise maximal among all links entering $\{v,w\}$. See Fig. 4 for an example.

Using that $\mathcal {L}$ is a laminar family, we can observe the following.

#### Claim

If $\ell$ is a link entering a terminal $t\in T$ and $\ell _{\max }$ is a maximal link entering *t*, then $\mathcal {C}^{\ell } \subseteq \mathcal {C}^{\ell _{\max }}$. Similarly, if $\ell$ is a link entering $\{v,w\}\in M$ and $\ell _{\max }$ is a maximal link entering $\{v,w\}$, then $\mathcal {C}^{\ell } \subseteq \mathcal {C}^{\ell _{\max }}$.

#### Proof

Let $\ell$ be a link entering a terminal $t\in T$ and $\ell _{\max }$ a maximal link entering *t*. Because $\mathcal {L}$ is a laminar family, the cuts of $\mathcal {L}$ that contain *t* form a chain $C_1\subsetneq C_2 \subsetneq \ldots \subsetneq C_q$. Thus, $\mathcal {C}^\ell$ is a prefix of that chain, i.e., $\mathcal {C}^\ell =\{C_1, C_2, \ldots , C_i\}$ for some $i\in [q]$. As $\ell _{\max }$ is a maximal link entering *t*, the prefix of the chain of *t* must be the largest one among all links, and thus $\mathcal {C}^{\ell _{\max }} \supseteq \mathcal {C}^{\ell }$, as desired.

The second part of the statement follows by an analogous reasoning. More precisely, let $\{v,w\}\in M$, let $\ell$ be a link entering $\{v,w\}$, and $\ell _{\max }$ be a maximal link entering $\{v,w\}$. Any cut $C\in \mathcal {L}$ fulfills either $\{v,w\}\subseteq C$ or $\{v,w\}\cap C = \emptyset$, because $\mathcal {L}\subseteq \mathcal {C}^M$ contains only cuts that are not covered by the matching *M*. Hence, all cuts in $\mathcal {C}^\ell$ are cuts of $\mathcal {L}$ containing both *v* and *w*. As before, the family of all cuts in $\mathcal {L}$ that contain both *v* and *w* form a chain because $\mathcal {L}$ is laminar. Because $\ell _{\max }$ is a maximal link entering $\{v,w\}$, the chain $\mathcal {C}^{\ell _{\max }}$ must be the largest one among all those links. Thus, $\mathcal {C}^{\ell _{\max }}\supseteq \mathcal {C}^{\ell }$, as claimed.

$$\square $$

We now explain how we construct a cheap feasible solution $x^M$ for LP (2). Let $T^M\subseteq T$ be the set of leaves of *G* that are not covered by the matching *M*. Then $|T^M| = |T| -2 |M|$. We define the vector $x^M$ as follows:

- for each leaf $t\in T^M$, we choose a maximal link $\ell \in \vec {L}$ entering *t* and set $x^M_{\ell } :=1$;
- for each in-link $\{v,w\}\in M_\mathrm{{in}}$, we choose a maximal link $\ell \in \vec {L}$ entering $\{v,w\}$ and set $x^M_{\ell } :=\tfrac{1}{2}$;
- for all other links in $\vec {L}$, we set $x^M_{\ell } :=0$.

Clearly, $x^M(L) = |T^M| + \tfrac{1}{2} |M_{\textrm{in}}| = \tfrac{1}{2} |M_{\textrm{in}}| + (|T| -2 |M|)$. Thus, it only remains to prove $x^M\bigl (\delta ^-_{\vec {L}}(C)\bigr ) \ge 1$ for all cuts $C\in \mathcal {L}$.

To this end, fix a cut $C\in \mathcal {L}$, and let $T_C \subseteq T\cap C$ be the set of terminals in *C* that have an incident link covering *C*. We first observe that if $T_C$ contains the head of a cross-link $\ell \in M_{\textrm{cross}}$, then, because $x^M_\ell =1$ and any cross-links covers all cuts containing its head, we have $x^M(\delta ^-_{\vec {L}}(C)) \ge 1$ as desired.

We now distinguish two cases. First, suppose $T_C$ contains a terminal $t\in T^M$ that is not covered by the matching *M*. Then we have $x_{\ell _{\max }}=1$ for a maximal link $\ell _{\max } \in \vec {L}$ entering *t*. Because $t\in T_C$, there exists a link $\ell \in \vec {L}$ that enters *t* and covers *C*. By the claim, the maximality of $\ell _{\max }$ implies that also $\ell _{\max }$ covers *C*. Hence, $x^M(\delta ^-_{\vec {L}}(C)) \ge x^M_{\ell _{\max }} = 1$.

We now consider the remaining case where all terminals in $T^C$ are covered by in-links in the matching *M*. Let $t_1 \in T^C \subseteq C$. (Note that $T_C$ is not empty because our CacAP instance is feasible.) In the matching *M*, the terminal $t_1$ is covered by an in-link $\{s_1,t_1\} \in M_\mathrm{{in}}$. Because the link $\{s_1,t_1\}$ is not bad, there exists a terminal $t_2 \in T_C {\setminus } \{s_1,t_1\}$, which is covered in the matching *M* by an in-link $\{s_2,t_2\}\in M_\mathrm{{in}}$. For each $i\in \{1,2\}$, we chose a maximal link $\ell _i\in \vec {L}$ entering $\{s_i,t_i\}$ and defined $x_{\ell _i}:=\tfrac{1}{2}$. Because $t_i\in T_C$, there exists a link $\ell \in \vec {L}$ that enters $t_i$ and covers *C*. By the claim, this implies that also the link $\ell _i$ covers the cut *C*. Hence, we have $x^M(\delta ^-_{\vec {L}}(C)) \ge x^M_{\ell _1} + x^M_{\ell _2} = \tfrac{1}{2} + \tfrac{1}{2} = 1$. $\square$

We now use Lemma 14 to show that (dir-LP) has a cheap feasible solution even if $\mathcal {C}^M$ is not necessarily laminar. This will complete the proof of Lemma 10.

#### Lemma 15

The optimum value of (dir-LP) is at most $\frac{1}{2}|M_{\textrm{in}}| + |T| -2 |M|$.

#### Proof

We consider the dual of (dir-LP), which is

3 $$\begin{aligned} \max \left\{ \sum _{C\in \mathcal {C}^M} \lambda _C \, \ \lambda \in \mathbb {R}_{\ge 0}^{\mathcal {C}^M},\ \sum _{C\in \mathcal {C}^M} \lambda _C \cdot \chi ^{\delta ^-_{\vec {L}}(C)} \le \chi ^{\vec {L}} \right\} . \end{aligned}$$

Suppose by the sake of deriving a contradiction that the optimum value of the primal LP (dir-LP) is strictly greater than $\frac{1}{2}|M_\mathrm{{in}}| + |T| -2 |M|$. Then, by strong duality, there exists a feasible solution $\lambda ^*$ to the dual LP (3) with

$$\begin{aligned} \sum _{C \in \mathcal {C}^M} \lambda ^*_C \ >\ \frac{1}{2}|M_{\textrm{in}}| + |T| -2 |M|. \end{aligned}$$

We will show by a standard combinatorial uncrossing argument that we may assume the support $\{C\in \mathcal {C}^M: \lambda ^*_C > 0\}$ of $\lambda ^*$ to be a laminar family $\mathcal {L}\subseteq \mathcal {C}^M$. Then by restricting $\lambda ^*$ to its support, we obtain a feasible solution to

4 $$\begin{aligned} \max \left\{ \sum _{C\in \mathcal {L}} \lambda _C \, \ \lambda \in \mathbb {R}_{\ge 0}^{\mathcal {L}},\ \sum _{C\in \mathcal {L}} \lambda _C \cdot \chi ^{\delta ^-_{\vec {L}}(C)} \le \chi ^{\vec {L}} \right\} \end{aligned}$$

with value $\sum _{C \in \mathcal {L}} \lambda ^*_C \ >\ \frac{1}{2}|M_{\textrm{in}}| + |T| -2 |M|$. This implies, again by duality, that the optimum value of the LP

$$\begin{aligned} \min \Bigl \{ x(\vec {L}): x\in \mathbb {R}^{\vec {L}}_{\ge 0}, x\bigl (\delta ^-_{\vec {L}}(C)\bigr ) \ge 1 \text { for all }C\in \mathcal {L}\Bigr \} \end{aligned}$$

is strictly greater than $\frac{1}{2}|M_\mathrm{{in}}| + |T| -2 |M|$, contradicting Lemma 14.

It remains to show that we may assume the support of $\lambda ^*$ to be laminar. Let $S, T \in \mathcal {C}^M$ be two crossing sets. Then

$$\begin{aligned} \chi ^{\delta ^-_{\vec {L}}(S)}+ \chi ^{\delta ^-_{\vec {L}}(T)} \ge \chi ^{\delta ^-_{\vec {L}}(S\cap T)}+\chi ^{\delta ^-_{\vec {L}}(S\cup T)}. \end{aligned}$$

Hence, if *S* and *T* are in the support of $\lambda ^*$, we can decrease $\lambda ^*_S$ and $\lambda ^*_T$ by some sufficiently small $\varepsilon >0$ and at the same time increase $\lambda ^*_{S\cap T}$ and $\lambda ^*_{S\cup T}$ by $\varepsilon$, such that the vector $\lambda ^*$ remains feasible for (3) and the value of $\sum _{C \in \mathcal {C}^M} \lambda ^*_C$ does not change. However, the value of $\sum _{C \in \mathcal {C}^M} \lambda ^*_C \cdot |C|^2$ increases. Therefore, if we choose $\lambda \in \mathbb {R}^{\mathcal {C}^M}_{\ge 0}$ to be a feasible solution to (3) with $\sum _{C \in \mathcal {C}^M} \lambda _C = \sum _{C \in \mathcal {C}^M} \lambda ^*_C$ that maximizes $\sum _{C \in \mathcal {C}^M} \lambda _C \cdot |C|^2$ among all such vectors $\lambda$, then the support of $\lambda$ is a laminar family $\mathcal {L}\subseteq \mathcal {C}^M$. $\square$

### Existence of good matching (proof of Lemma 11)

We now provide a proof of Lemma 11, which shows that there is a good matching *M*.

Let $H\subseteq L$ be any solution of the given Leaf-to-Leaf+ CacAP instance $(G=(V,E),L)$. Let $M^{H}\subseteq H$ be an inclusion-wise maximal matching consisting only of leaf-to-leaf links in *H* that are not bad. We denote by $T_{\text {cov}}\subseteq T$ the set of leaves covered by $M^{H}$. Notice that each link $\ell \in H \setminus M^{H}$ satisfies either

(i) one endpoint of $\ell$ belongs to $T_{\text {cov}}$; or
(ii) one endpoint of $\ell$ coincides with the root *r*; or
(iii) $\ell$ is a bad link.

Let $H^{\textrm{bad}}\subseteq H$ be the set of bad links in *H* for which 1. does not hold. The following claim upper bounds the number of links in $H^{\textrm{bad}}$.

#### Lemma 16

$$\begin{aligned} \sum _{v\in T\setminus T_{\textrm{cov}}} |\delta _{H}(v)| \ge |T\setminus T_{\textrm{cov}}| + |H^{\textrm{bad}}|. \end{aligned}$$

Before proving Lemma 16, we show that it implies the desired result. Assuming Lemma 16, we get

5 $$\begin{aligned} \begin{aligned} |H|&= |M^{H}| + |H \setminus M^{H}| \\&\ge |M^{H}| + \sum _{v\in T\setminus T_{\textrm{cov}}} |\delta _{H}(v)| - |H^{\textrm{bad}}| \\&\ge |M^{H}| + |T\setminus T_{\textrm{cov}}| \\&= |M^{H}| + \left( |T|-2|M^{H}|\right) , \end{aligned} \end{aligned}$$

where the first inequality holds because all links in *H* that are incident to a vertex in $T\setminus T_\mathrm{{cov}}$ are contained in $H \setminus M^{H}$ and only links in $H^{\textrm{bad}}$ have both endpoints in $T\setminus T_\mathrm{{cov}}$, and the second inequality follows from the claim. Hence, if $M\subseteq L$ is a matching using only leaf-to-leaf links that are not bad and such that it minimizes $|M| + \tfrac{1}{2}|M_{\textrm{in}}|+(|T|-2|M|)$ among all such matchings, we obtain

$$\begin{aligned} |M|+\frac{1}{2}|M_{\textrm{in}}|+(|T| - 2|M|) \le |M^{H}|+\frac{1}{2}|M^{H}_{\textrm{in}}|+\left( |T| - 2|M^{H}|\right) \le |H| + \frac{1}{2}|H_{\textrm{in}}|, \end{aligned}$$

where the first inequality follows from the fact that *M* has been chosen to minimize that expression and the second one is due to (5) and $\frac{1}{2}|M^{H}_{\textrm{in}}| \le \frac{1}{2}|\textrm{H}_{\textrm{in}}|$, which holds because $M^{H}_{\textrm{in}} \subseteq H_{\textrm{in}}$.

It remains to prove Lemma 16.

#### Proof of Lemma 16

Let $G^{\textrm{bad}}= (T{\setminus } T_{\textrm{cov}},H^{\textrm{bad}})$ be the graph induced by links in $H^{\textrm{bad}}$ on $T\setminus T_{\textrm{cov}}$. Let $\mathcal {K}=(T_{\mathcal {K}}, H^\mathrm{{bad}}_{\mathcal {K}})$ be one of its connected components and let $n_{\mathcal {K}}:=|T_{\mathcal {K}}|$ and $m_{\mathcal {K}}:=|H^\mathrm{{bad}}_{\mathcal {K}}|$ denote the number of vertices and edges of $\mathcal {K}$, respectively. We will show

6 $$\begin{aligned} \sum _{v\in T_{\mathcal {K}}} |\delta _{H}(v)| \ge n_{\mathcal {K}} + m_{\mathcal {K}}. \end{aligned}$$

The claim then follows by summing over all connected components of $G^\mathrm{{bad}}$.

For each link $\ell \in H^\mathrm{{bad}}_{\mathcal {K}}$, there exists a cut $C^{\ell }\in \mathcal {C}$ such that $\ell =\{u,v\}$ is a bad link with respect to $C^{\ell }$. Let

7 $$\begin{aligned} C^{\mathcal {K}} :=\bigcup _{\ell \in H^\mathrm{{bad}}_{\mathcal {K}}} C^{\ell }. \end{aligned}$$

Then $C^{\mathcal {K}}$ is also a 2-cut in *G*, which can be derived via well-known combinatorial uncrossing arguments as follows. Start with the family $\mathcal {F}:=\{C^{\ell } :\ell \in H_{\mathcal {K}}^{\textrm{bad}}\}$. We maintain that $\mathcal {F}$ is a family of 2-cuts that are *connected* in the sense that the graph with vertex set $\mathcal {F}$ that has an edge between $C_1\in \mathcal {F}$ and $C_2\in \mathcal {F}$ if $C_1\cap C_2 \ne \emptyset$, is connected. Because $\mathcal {K}$ is connected, this holds for the starting $\mathcal {F}$. We then successively select any two sets $C_1,C_2\in \mathcal {F}$ with $C_1\cap C_2\ne \emptyset$ and replace it by $C_1\cup C_2$. Because $C_1$ and $C_2$ are 2-cuts that intersect (and both do not contain the root *r*), also $C_1\cup C_2$ is a 2-cut. (This is the step implied by classic uncrossing techniques; see, e.g., Lemma 24 in [3].) At the end of this procedure we have the single cut $C^{\mathcal {K}}$ in our family, which is therefore also a 2-cut, as desired.

Because *H* is a Leaf-to-Leaf+ CacAP solution, there exists a link $\ell _{\mathcal {K}}$ in *H* that covers the 2-cut $C^{\mathcal {K}}$. Note that one endpoint of $\ell _{\mathcal {K}}$ must be contained in $T_{\mathcal {K}}$ because by (7) we have

$$\begin{aligned} \delta _H(C^{\mathcal {K}}) \subseteq \bigcup _{\ell \in H^{\textrm{bad}}_{\mathcal {K}}} \delta _H(C^{\ell }), \end{aligned}$$

and the choice of $C^{\ell }$ together with the definition of bad links implies that for $\ell =\{u,v\}\in H^{\textrm{bad}}$, any link in $\delta _H(C^{\ell })$ must have $u\in T_{\mathcal {K}}$ or $v\in T_{\mathcal {K}}$ as one of its endpoints. Moreover, one endpoint of $\ell _{\mathcal {K}}$ is not contained in $T_{\mathcal {K}}$, because $T_{\mathcal {K}}\subseteq C^{\mathcal {K}}$. Hence, in particular, $\ell _{\mathcal {K}}$ is not contained in the set $H^\mathrm{{bad}}_{\mathcal {K}}$ of links of the component $\mathcal {K}$. Because each of the $m_{\mathcal {K}}$ many links in $H^\mathrm{{bad}}_{\mathcal {K}}$ has both endpoints in $T_{\mathcal {K}}$ and the link $\ell _{\mathcal {K}}$ has only one endpoint in $T_{\mathcal {K}}$, we have

$$\begin{aligned} \sum _{v\in \mathcal {K}} |\delta _{H}(v)| \ge 2m_{\mathcal {K}} +1 \ge n_{\mathcal {K}} + m_{\mathcal {K}}, \end{aligned}$$

because $\mathcal {K}$ is connected. This shows (6).

## Reducing to $\mathbf {O(1)}$-wide instances

Recall that [3] gave a reduction from general instances of CacAP to *O*(1)-*wide instances* Definition 3). In this section we show that this reduction can be adapted for the case of leaf-to-leaf CacAP instances: we show that, at the expense of an arbitrarily small constant loss in the approximation factor, it suffices to consider *O*(1)-wide leaf-to-leaf CacAP instances.

### Theorem 4

Let $\alpha \ge 1$, $\varepsilon > 0$, and $k:=\frac{256(16+3\varepsilon )}{\varepsilon ^3}$. Given an $\alpha$-approximation algorithm $\mathcal {A}$ for *k*-wide Leaf-to-Leaf CacAP instances, there is an $\alpha \cdot (1+\varepsilon )$-approximation algorithm $\mathcal {B}$ for (unrestricted) Leaf-to-Leaf CacAP that calls $\mathcal {A}$ at most polynomially many times and performs further operations taking polynomial time.

The reason why we cannot use the reduction to *O*(1)-wide instances given in [3] as a black box is that in our setting we have to make sure that we maintain the property that all links are leaf-to-leaf links. The *k*-wide instances that result from the reduction given in [3] are obtained from the original instance by (possibly repeatedly) deleting links and applying the following two types of operations, which we call *splitting* and *contraction*. Splitting on a 2-cut allows for decomposing a CacAP instance into smaller independent ones, at a limited cost under some well-defined circumstances.

### Definition 17

(splitting) Let $C \subseteq V$ with $|\delta _E(C)|=2$. *Splitting at* *C* leads to two sub-instances $\mathcal {I}_C$ and $\mathcal {I}_{V\setminus C}$, where $\mathcal {I}_C$ is the instance obtained from $\mathcal {I}$ by contracting all vertices except for those in *C*, and $\mathcal {I}_{V\setminus C}$ is the instance obtained from $\mathcal {I}$ by contracting *C*.

Contraction operations are the operations used in defining residual instances (see Definition 12). Hence, they can be used to fix some links in a solution and obtain the residual CacAP instance capturing how the fixed links can be complemented to a solution to the original CacAP instance.

### Definition 18

(contraction) Let $\mathcal {I}=(G=(V,E),L)$ be a CacAP instance and let $\ell \in L$. *To contract* $\ell =\{u,v\}$ means that we contract the set of vertices that are contained in every *u*-*v* path in *G*.

In [3], contraction operations are used to fix in the solution a cheap set of links covering all 2-cuts on which splitting might be expensive. Subsequently, in the thus obtained residual instance, splitting operations can be performed to obtain an *O*(1)-wide instance.

We observe that splitting operations maintain the leaf-to-leaf property.

### Lemma 19

Let $\mathcal {I}=(G=(V,E),L)$ be an instance of CacAP and let $C \subseteq V$ with $|\delta _E(C)|=2$. If $\mathcal {I}$ is an instance of Leaf-to-Leaf CacAP, then also the CacAP instances $\mathcal {I}_C$ and $\mathcal {I}_{V\setminus C}$ that result from splitting $\mathcal {I}$ at *C* are instances of Leaf-to-Leaf CacAP.

More generally, if *p* is the number of vertices in $\mathcal {I}$ that are endpoints of a link in *L*, but are not leaves of *G*, then the total number of such non-leaf endpoints of links in $\mathcal {I}_C$ and $\mathcal {I}_{V\setminus C}$ is also *p*.

### Proof

Let $p_C$ and $p_{V\setminus C}$ be the number of non-leaf endpoints of links in $\mathcal {I}$ that are contained in *C* and $V\setminus C$, respectively. Then $p_C + p_{V\setminus C} = p$. In the sub-instance $\mathcal {I}_C$, the set $V\setminus C$ is contracted into a single vertex $v_{V\setminus C}$ that is a leaf for the new instance, because $|\delta _E(C)| = |\delta _E(v_{V{\setminus } C})| =2$. For each link $\ell \in \delta _E(C)$, one of its endpoints is replaced by the leaf $v_{V\setminus C}$ while the other endpoint does not change. As the endpoints of other links are not changed and all leaves of *G* in *C* remain leaves after the contraction of $V\setminus C$, the number of non-leaf endpoints of links in $\mathcal {I}_C$ is $p_C$. By symmetry, it follows that the number of non-leaf endpoints of links in $\mathcal {I}_{V\setminus C}$ is $p_{V\setminus C}$.

$$\square $$

**Fig. 5:**
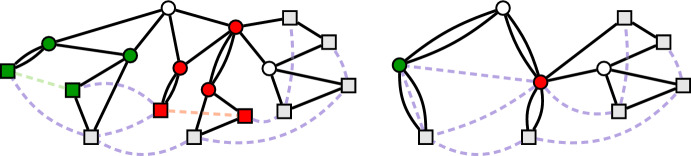
The left picture shows a leaf-to-leaf instance where two links to be contracted are highlighted in green and red, respectively, together with the vertices to be contracted. The right picture shows the resulting instance after the two links have been contracted. Notice that after these contraction operations there exist both leaf-to-non-leaf links and non-leaf-to-non-leaf links

Unfortunately, contractions of links do not maintain the leaf-to-leaf property. See Fig. 5. However, we will show that such contractions are not applied too often.

The following definition formally captures the type of decompositions that we obtain from the reduction from [3].

### Definition 20

Let $\mathcal {I}=(G=(V,E),L)$ be a CacAP instance. A sequence of splitting operations, contraction operations, and deletions of links is called a $(\gamma ,k)$-*splitting of (G, L)* if the following two properties are satisfied.

- All instances obtained after applying the sequence of operations are *k*-wide; we call these instances the *split-minors* of the $(\gamma ,k)$-splitting.
- The number of contraction operations is at most $\gamma$.

The proof from [3] yields the following general reduction statement.

### Theorem 21

([3]) Let $f: \{ \mathcal {I}: \mathcal {I}\text { CacAP instance }\} \rightarrow \mathbb {R}_{\ge 0}$ be a polynomial-time computable function, let $\varepsilon > 0$ and let $k:=\frac{64(8+3\varepsilon )}{\varepsilon ^3}$. Suppose we are given an efficient algorithm $\mathcal {A}$ that for any feasible *k*-wide CacAP instance $\mathcal {I}$ computes a solution with at most $\alpha \cdot \textrm{OPT}(\mathcal {I})+f(\mathcal {I})$ many links. Then there is an algorithm $\mathcal {B}$ that for any feasible CacAP instance $\mathcal {I}$ computes a solution with at most

$$\begin{aligned} \alpha \cdot (1+\varepsilon )\cdot \textrm{OPT}(\mathcal {I})\quad +\ \max _{\begin{array}{c} \mathcal {S}\!\text { an }(\varepsilon |\textrm{OPT}(\mathcal {I})|,k)-\\ \text {splitting of }\mathcal {I} \end{array}}\quad \sum _{\mathcal {J}\!\text { split minor of }\mathcal {S}} f(\mathcal {J}) \end{aligned}$$

many links, while calling $\mathcal {A}$ at most polynomially many times and performing further operations taking polynomial time.

Because [3] does not formally state Theorem 21 in this form, we provide details on how the theorem follows from [3] in Appendix A. Observe that when $f\equiv 0$, the statement of Theorem 21 gives a reduction to *k*-wide instances similar to Theorem 4, but for general (not necessarily leaf-to-leaf) CacAP instances. In order to prove Theorem 4, we will apply Theorem 21 with the function *f* being defined by

8 $$\begin{aligned} \begin{aligned} f\bigl ((G=(V,E),L)\bigr ) :=\bigl | \bigl \{&v\in V: v\text { is not a leaf of }G, \\&\text {but an endpoint of a link in }L\bigr \}\bigr |. \\ \end{aligned} \end{aligned}$$

The next lemma shows why this choice of the function *f* is useful for proving Theorem 4.

### Lemma 22

Let the function *f* be defined by (8). Then, for any Leaf-to-Leaf CacAP instance $\mathcal {I}$, we have

$$\begin{aligned} \max _{\begin{array}{c} \mathcal {S}\!\text { an }(\gamma ,k)-\\ \text {splitting of }\mathcal {I} \end{array}}\quad \sum _{\mathcal {J}\!\text { split minor of }\mathcal {S}} f(\mathcal {J}) \ \le \ \gamma . \end{aligned}$$

### Proof

Contracting a single link increases the number of non-leaf endpoints of links by at most one. Moreover, the deletion of links does not increase the total number of non-leaf endpoints and by Lemma 19 the same holds for splitting operations. Thus, for every $(\gamma , k)$-splitting $\mathcal {S}$, the total number $\sum _{\mathcal {J}\!\text { split minor of }\mathcal {S}} f(\mathcal {J})$ of non-leaf endpoints is upper bounded by the number of contraction operations in the splitting $\mathcal {S}$, which is at most $\gamma$ by Definition 20.

$$\square $$

In order to be able to prove Theorem 4 using Theorem 21, we will show that using an approximation algorithm for Leaf-to-Leaf CacAP, we can give an approximation algorithm for CacAP with few non-leaf endpoints of links. More precisely, we show the following lemma.

### Lemma 23

Let $\alpha \ge 1$ and the function *f* be defined by (8). Suppose we are given an $\alpha$-approximation algorithm $\mathcal {A}$ for *k*-wide Leaf-to-Leaf CacAP instances. Then there is an algorithm $\mathcal {A}'$ that for any feasible *k*-wide CacAP instance $\mathcal {I}$ computes a solution with at most $\alpha \cdot \textrm{OPT}(\mathcal {I}) + f(\mathcal {I})$ many links, while calling $\mathcal {A}$ at most once and performing further operations taking polynomial time.

### Proof

Given a feasible *k*-wide CacAP instance $\mathcal {I}=(G=(V,E),L)$, we will transform it into a *k*-wide leaf-to-leaf instance. As a first step, we will compute a set *X* of at most $f(\mathcal {I})$ many links that we can contract to make sure that every non-leaf endpoint of a link in $\mathcal {I}$ is merged with a leaf of *G* or has no incident links anymore. In a second step, we will then modify the resulting CacAP instance $\mathcal {I}^X$ such that we obtain a *k*-wide leaf-to-leaf instance $\tilde{\mathcal {I}}^X$ with the following properties:

- $$|\textrm{OPT}(\tilde{\mathcal {I}}^X)| \le |\textrm{OPT}(\mathcal {I})|$$, and
- for every solution *F* of $\tilde{\mathcal {I}}^X$, the link set $F\cup X$ is a feasible solution for the instance $\mathcal {I}$.

Finally, we use the given algorithm $\mathcal {A}$ to compute an $\alpha$-approximate solution *F* for the *k*-wide Leaf-to-Leaf CacAP instance $\tilde{\mathcal {I}}^X$. Then, using $|X|\le f(\mathcal {I})$ and properties (a) and (b), we can conclude that $F\cup X$ is a solution for $\mathcal {I}$ with

$$\begin{aligned} |F\cup X| \le \alpha \cdot \left| \textrm{OPT}\left( \tilde{\mathcal {I}}^X\right) \right| + |X| \le \alpha \cdot |\textrm{OPT}(\mathcal {I})| + f(\mathcal {I}). \end{aligned}$$

Let us now explain how we choose the set $X\subseteq L$ and construct the instance $\mathcal {I}^X$. We will choose a set $X\subseteq L$ with $|X| \le f(\mathcal {I})$; then we define $\mathcal {I}^X$ to be the residual instance of $\mathcal {I}$ with respect to *X* (see Definition 12). It is well known that $F\subseteq L$ is a feasible solution to the residual instance $\mathcal {I}^X$ if and only if $F\cup X$ is a feasible solution to the original instance (see for example Corollary 20 in [3]). We will call a node of the residual instance that arose from the contraction of several vertices of the original instance $\mathcal {I}$, a *supernode*. We say that the vertices that were contracted to obtain a supernode *s* *belong to* *s*.

Let $B:=\left\{ v\in V: v\text { is not a leaf of }G, \text { but an endpoint of a link in }L\right\}$. Then $f(\mathcal {I}) = |B|$. The following claim describes how we choose the set *X* of links that we will contract.

### Claim

We can in polynomial time find a set $X\subseteq L$ with $|X|\le f(\mathcal {I})$ such that the residual instance with respect to *X* has the following properties:

(i) every vertex $v\in B$ belongs to a supernode, and
(ii) for every supernode *s*
  there is a leaf of *G* that belongs to *s*, or
  *s* has no incident link in the residual instance $\mathcal {I}^X$.

### Proof

We construct the set *X* by the following algorithm.

1. Initialize $$X=\emptyset $$.
2. As long as (i) or (ii) is not fulfilled, iterate the following:
  Choose *v* to be either a vertex in *B* violating (i) or a supernode violating (ii).
  Choose an arbitrary link $\ell \in \delta _L(v)$ and add $\ell$ to *X*.
  Apply the contraction operation for $\ell$ (Definition 18).

Note that a link $\ell \in \delta _L(v)$ exists in every iteration of the algorithm. If $v\in B$, this follows from the definition of *B*, and if *v* is a supernode, this follows from the fact that *v* violates (ii). At the end of the algorithm, the instance $\mathcal {I}^X$ fulfills (i) and (ii) by construction. In order to prove $|X|\le f(\mathcal {I}) = |B|$, we show that the number of vertices violating (i) or (ii) strictly decreases in every iteration of the algorithm. This will conclude the proof because at the beginning of the algorithm (i) and (ii) are violated only by the vertices in *B* (as no supernodes exist). Now consider an iteration in which we chose a vertex *v* violating (i) or (ii) and contracted $\ell =\{v,w\}$. If *w* is a leaf of *G* or a supernode with a leaf of *G* belonging to *w*, then the supernode arising from the contraction of $\ell$ does not violate (ii). Otherwise, by the definition of *B*, the vertex *w* must be either an element of *B* (violating (i)) or a supernode violating (ii). When contracting $\ell$, the two vertices *v* and *w* violating  (i) or (ii) get merged into a single supernode. In any of these cases the number of vertices violating (i) or (ii) decreases strictly.

$$\square $$

It remains to show that we can transform the residual instance $\mathcal {I}^X$ into a *k*-wide instance of Leaf-to-Leaf CacAP with properties (a) and (b). We construct the instance $\tilde{\mathcal {I}}^X$ from $\mathcal {I}^X$ as follows. For every supernode *s* that has at least one incident link in $\mathcal {I}^X$, we add a new auxiliary vertex $t_s$ and two copies of the edge $\{s,t_s\}$ to the cactus. Then we still have a cactus and $t_s$ is one of its leaves. Moreover, we replace every link $\{s, v\}$ by the link $\{ t_s, v\}$. The leaf-to-leaf instance that results from applying this transformation for all supernodes with at least one incident link is the instance $\tilde{\mathcal {I}}^X = (\tilde{G}, \tilde{L})$. We view $\tilde{L}$ as a subset of *L* by identifying each link in $\tilde{L}$ with the corresponding link in *L* from which it arose in the construction. We now show that $\tilde{\mathcal {I}}^X$ has the desired properties.

### Claim

For every solution *F* of $\tilde{\mathcal {I}}^X$, the link set $F\cup X$ is a feasible solution for the instance $\mathcal {I}$.

### Proof

Let $(G^X=(V^X,E^X), L^X) :=\mathcal {I}^X$ and let *F* be a solution of $\tilde{\mathcal {I}}^X = (\tilde{G} = (\tilde{V}, \tilde{E}), \tilde{L})$. Then the graph $(\tilde{V}, \tilde{E} \cup F)$ is 3-edge-connected. Because $(V^X, E^X \cup F)$ arises from this graph by contracting the pair $\{s, t_s\}$ for every supernode $s \in V^X$ for which we added a vertex $t_s$, also the graph $(V^X, E^X \cup F)$ is 3-edge connected, i.e., *F* is a feasible solution for $\mathcal {I}^X$. Because $\mathcal {I}^X$ is the residual instance of $\mathcal {I}$ with respect to *X*, we indeed have that $F\cup X$ is a solution for $\mathcal {I}$ (see for example [3, Corollary 20]).

$$\square $$

### Claim

The instance $\tilde{\mathcal {I}}^X$ is feasible and we have $|\textrm{OPT}(\tilde{\mathcal {I}}^X)| \le |\textrm{OPT}(\mathcal {I})|$.

### Proof

We construct from $\textrm{OPT}(\mathcal {I})$ a solution *F* for $\tilde{\mathcal {I}}^X$ of at most the same cardinality. The solution *F* that we construct contains for every link $\ell =\{v,w\} \in \textrm{OPT}(\mathcal {I})$

(i) the link $\ell$ if *v* and *w* do not belong to the same supernode, and
(ii) an arbitrary link incident to $t_s$ if *v* and *w* belong to the same supernode *s* and *s* has an incident link in $\mathcal {I}^X$. (Note that in this case $t_s$ exists and has an incident link in $\tilde{\mathcal {I}}^X$.)

By construction $|F|\le |\textrm{OPT}(\mathcal {I})|$. Because $\textrm{OPT}(\mathcal {I})$ is a feasible solution for $\mathcal {I}$ and hence for $\mathcal {I}^X$, every violated cut, i.e., every 2-cut of the cactus $\tilde{G}$ that does not contain a link from *F*, must be of the form $\delta _{\tilde{E}}(t_s)$ for some supernode *s*.

Suppose such a violated cut exists. The construction of $\tilde{\mathcal {I}}^X$ implies that there exists a link that is incident to $t_s$. Moreover, by the first claim this implies that there is a leaf *v* of *G* that belongs to the supernode *s*. The solution $\textrm{OPT}(\mathcal {I})$ must contain a link $\ell$ that is incident to *v*. If both endpoints of $\ell$ belong to the supernode *s*, then *F* contains a link in $\delta _{\tilde{L}}(t_s)$ by (ii). Otherwise, $\ell \in F \cap \delta _{\tilde{L}}(t_s)$, because we replaced the endpoint *s* of $\ell$ by $t_s$ in the construction of $\mathcal {I}^X$. Thus, $\delta _{\tilde{E}}(t_s)$ is not a violated cut, a contradiction. $\square$

### Claim

The instance $\tilde{\mathcal {I}}^X$ is *k*-wide.

### Proof

Let *r* be a *k*-wide root of *G* and let $\tilde{r}$ be either *r* or the supernode in $\tilde{G}$ to which *r* belongs. We show that $\tilde{r}$ is a *k*-wide root for $\tilde{G}$. To this end, let us consider the vertex set $\tilde{W}$ of a connected component of $\tilde{G} - \tilde{r}$. Then the vertices in *V* that are contained in $\tilde{W}$ or belong to a supernode contained in $\tilde{W}$ are all part of the same connected component of $G - r$. Hence, at most *k* of these vertices are leaves of *G*. To complete the proof, we will show that every leaf of $\tilde{G}$ in $\tilde{W}$ is either a leaf of *G* or an auxiliary vertex $t_s$ for a supernode *s* with a leaf of *G* belonging to *s*.

Let $\tilde{t} \in \tilde{W}$ be a leaf of $\tilde{G}$. If $\tilde{t}$ is a vertex in *V*, i.e., it is neither a supernode nor an auxiliary vertex, then its degree in $\tilde{G}$ is the same as in *G*, implying that $\tilde{t}$ is a leaf of *G*. If $\tilde{t}$ is an auxiliary vertex $t_s$ for a supernode *s*, then by the first claim, there exists a leaf of *G* that belongs to *s*. Finally, suppose $\tilde{t}$ is a supernode. Then $\tilde{t}$ cannot have any incident links as otherwise we would have added the leaf $t_s$ incident to $\tilde{t}$. However, because $\tilde{t}$ is a leaf of $\tilde{G}$, this contradicts the feasibility of the instance $\tilde{\mathcal {I}}^X$ (previous claim).

$$\square $$

The claims above imply that $\tilde{\mathcal {I}}^X$ indeed has the desired properties. $\square$

Theorem 4 now follows directly from Theorem 21, Lemma 22, and Lemma 23.

## Combining the matching-based approach with the stack analysis (Proof of Theorem 1)

To obtain approximation factors below $$\tfrac{4}{3}$$, we use the stack analysis approach from [3], which strengthens the procedure guaranteed by Lemma 5. In this section we explain how we can adapt this stack analysis approach from [3] for our purposes and we prove that it leads to the claimed approximation ratio of $$1.29 $$.

Let us first recall the definition of *shadows* and *minimality* of links from [3].

### Definition 24

Let (*G*, *L*) be a CacAP instance and let $\{u,v\}$ be a link. Then  is a *shadow* of the link $\{u,v\}$ if $\bar{u}$ and $\bar{v}$ are vertices that lie on every *u*-*v* path in *G*. A shadow $\bar{\ell }$ of link $\ell$ is a *strict shadow* of $\ell$ if $\bar{\ell }\ne \ell$.

Following [3], we say that a link $\ell _1\in L$ is *minimal with respect to* $\ell _2\in L$ if for any strict shadow  of $\ell _1$, the 2-cuts covered by $\{\bar{\ell _1},\ell _2\}$ are a strict subset of those covered by $\{\ell _1,\ell _2\}$ and the 2-cuts covered by $\{\ell _2\}$ are a strict subset of those covered by $\{\ell _1,\ell _2\}$; or formally, for any strict shadow $\bar{\ell _1}$ of $\ell _1$,

$$\begin{aligned} \begin{aligned} \{ C\in \mathcal {C}: \{\bar{\ell }_1,\ell _2\} \cap \delta _L(C) \ne \emptyset \} \subsetneq&\ \{ C \in \mathcal {C}: \{\ell _1, \ell _2\} \cap \delta _L(C) \ne \emptyset \},\text { and} \\ \{ C\in \mathcal {C}: \{\ell _2\} \cap \delta _L(C) \ne \emptyset \} \subsetneq&\ \{ C \in \mathcal {C}: \{\ell _1, \ell _2\} \cap \delta _L(C) \ne \emptyset \}. \end{aligned} \end{aligned}$$

We remark that the second of the two conditions above is implied by the first one whenever $\ell _1$ admits a strict shadow. However, for the case where $\ell _1$ does not admit a strict shadow, which corresponds to both endpoints of $\ell _1$ lying in the same cycle of the cactus, the second condition is necessary.

We now introduce the notion of *weakly* $L_\mathrm{{cross}}$-*minimality*. A similar notion, called $L_\mathrm{{cross}}$-minimality, has been introduced in [3]. The reason why we work with the notion of weak $L_\mathrm{{cross}}$-minimality is that we will need this later to combine the stack analysis approach with the matching-based approach from Sect. 2.

### Definition 25

A set $F\subseteq L$ is called weakly $L_{\textrm{cross}}$-minimal if for every two distinct links $\ell ,\ell '\in F_{\textrm{cross}}$ the link $\ell$ is minimal with respect to $\ell '$.

The property of $L_\mathrm{{cross}}$-*minimality* introduced in [3] requires in addition that every cross-link $\ell _1\in F_{\textrm{cross}}$ is minimal with respect to every in-link $\ell _2 \in F_{\textrm{in}}$. However, for leaf-to-leaf instances, the notion of weak $L_\mathrm{{cross}}$-minimality will be sufficient for the stack analysis approach.

The stack analysis approach first solves a linear program and then rounds the LP solution to a CacAP solution. We will next introduce the polytope over which this linear program optimizes.

For a *k*-wide CacAP instance (*G*, *L*) with root *r* let $G_1,\dots , G_p$ denote the principal subcacti. Moreover, for $i\in \{1,\dots ,p\}$, we define $L_i \subseteq L$ to be the set of links that have at least one endpoint in $G_i$ that is distinct from the root *r*. We define

$$\begin{aligned} P^{\min }(G_i,L_i) :=\textrm{conv}&\Bigg ( \Bigg \{ \chi ^F : F \subseteq L_i\text { is a weakly } L_{\textrm{cross}}\text { -minimal} \\&\qquad \quad \text {feasible solution for } G_i \Bigg \} \Bigg ) \end{aligned}$$

to be the convex hull of incidence vectors of weakly $L_{\textrm{cross}}$-minimal feasible CacAP solutions for $G_i$, where a feasible CacAP solution for $G_i$ is a set of links that covers every $C\in \mathcal {C}$ that is a subset of the vertices of $G_i$. A key polytope in our approach, over which we will optimize later (together with one additional constraint), is

$$\begin{aligned} P^{\textrm{min}}_{\textrm{bundle}}(G,L) :=\left\{ x\in [0,1]^L: x|_{L_i} \in P^{\min }(G_i,L_i) \text { for all }i\in \{1,\dots ,p\} \right\} . \end{aligned}$$

We first observe that we can optimize in polynomial time over $P^{\min }_{\textrm{bundle}}(G,L)$, as stated below. The proof is essentially identical to one used in [3] (to prove Lemma 42). For completeness, we repeat the proof below, adapted to our context.

### Lemma 26

If *G* is *k*-wide for some constant *k*, then one can optimize in polynomial time any linear function over $P^{\min }_{\textrm{bundle}}(G, L)$.

### Proof

We first show that for every $i\in \{1,\dots ,p\}$, we can optimize over $P^{\min }(G_i,L_i)$ in polynomial time when *k* is constant. To this end, we observe that any weakly $L_{\textrm{cross}}$-minimal solution for $(G_i,L_i)$ contains at most *k* cross-links. Indeed, any two cross-links of $L_i$ in a weakly $L_{\textrm{cross}}$-minimal solution must be incident to different leaves of $G_i$. Hence, we can “guess” all those cross-links in polynomial time by enumerating all possible choices. Then we can complete this set of cross-links in a cheapest possible way, using that instances of CacAP with a constant number of terminals are polynomial-time solvable [2].

This implies that we can also optimize in polynomial time over the polytope $P^{\min }_{\textrm{bundle}}(G, L)$, because being able to optimize in polynomial time over $P^{\min }(G_i,L_i)$ implies that we can separate in polynomial time over $P^{\min }(G_i,L_i)$ and thus also over $P^{\textrm{min}}_{\textrm{bundle}}(G,L)$.

$$\square $$

Note that in general, $P^{\textrm{min}}_{\textrm{bundle}}(G,L)$ is not a relaxation of the CacAP problem and it might even be the case that the polyope $P^{\textrm{min}}_{\textrm{bundle}}(G,L)$ is empty even though the instance (*G*, *L*) is feasible. The reason is that in general, not every instance (*G*, *L*) has a weakly $L_\mathrm{{cross}}$-minimal solution. However, we will show that every *root-shadow complete* instance of Leaf-to-Leaf+ CacAP has a weakly $L_\mathrm{{cross}}$-minimal optimum solution (see Lemma 28 below).

### Definition 27

An instance (*G*, *L*) of Leaf-to-Leaf+ CacAP is *root-shadow complete* if for every cross link $\{u,v\}\in L_\mathrm{{cross}}$, both $\{u,r\}$ and $\{r,v\}$ are contained in *L*. Then $\{u,r\}$ and $\{r,v\}$ are the *root shadows* of $\{u,v\}$.

We may always assume that the leaf-to-leaf+ instance that we are given is root-shadow complete because, given an arbitrary leaf-to-leaf+ instance (*G*, *L*) with root *r*, we can consider its *root-shadow completion*

$$\begin{aligned} \bigl (G,L \cup \bigl \{\{u,r\}, \{v,r\}: \{u,v\}\in L_\mathrm{{cross}}\bigr \}\bigr ) \end{aligned}$$

that we obtain by adding all root-shadows of cross-links. Given any solution to the root-shadow completion we can always turn it into a solution of the original instance with the same number of links by replacing every root shadow by the original link. We remark that the root-shadow completion of a leaf-to-leaf+ instance is again a leaf-to-leaf+ instance. However, the root-shadow completion of a pure leaf-to-leaf instance is not a leaf-to-leaf instance and this is the reason why we work with leaf-to-leaf+ instances in this paper.

The following lemma shows that each Leaf-to-Leaf+ CacAP instance has some optimal solution that is weakly $L_{\textrm{cross}}$-minimal. This justifies our usage of $P^{\textrm{min}}_{\textrm{bundle}}$ as a proxy for a relaxation, because Lemma 28 implies that for any root-shadow complete CacAP instance (*G*, *L*), we have $\min \{ x(L): x\in P^{\textrm{min}}_{\textrm{bundle}}(G,L) \} \le |\textrm{OPT}|$ because $\chi ^{\textrm{OPT}}\in P^{\textrm{min}}_{\textrm{bundle}}(G,L)$ for any weakly $L_\mathrm{{cross}}$-minimal optimum solution $\textrm{OPT}$.

### Lemma 28

Every root-shadow complete instance of Leaf-to-Leaf+ CacAP has a weakly $L_\mathrm{{cross}}$-minimal optimum solution.

### Proof

Let $\textrm{OPT}$ be an optimum solution of a Leaf-to-Leaf+ CacAP instance with a minimum number of cross-links among all optimum solutions. We claim that $\textrm{OPT}$ is weakly $L_\mathrm{{cross}}$-minimal. Suppose this is not the case. Then there exist cross-links $\ell _1,\ell _2 \in \textrm{OPT}_{\textrm{cross}}$ such that $\ell _1$ is not minimal with respect to $\ell _2$. Let $\ell _1=\{s,t\}$ and note that any strict shadow of $\ell _1$, i.e., any shadow $\bar{\ell _1}\ne \ell _1$ of $\ell _1$, covers at most one of the 2-cuts $\{s\},\{t\} \in \mathcal {C}$. Thus, one of the endpoints of $\ell _1$, say *t*, must be also an endpoint of $\ell _2$, because otherwise for every strict shadow $\bar{\ell }_1$ of $\ell _1$ at least one of the 2-cuts $\{s\},\{t\} \in \mathcal {C}$ would be uncovered by $\{\bar{\ell }_1,\ell _2\}$, even though it was covered by $\{\ell _1,\ell _2\}$. Because $\ell _1=\{s,t\}$ and $\ell _2$ are both cross-links and have the common endpoint *t*, the set of 2-cuts in $\mathcal {C}$ covered by $\{s,r\}$ and $\ell _2$ is the same as the set of 2-cuts covered by $\ell _1$ and $\ell _2$. Hence, we can replace the link $\ell _1$ in $\textrm{OPT}$ by its root-shadow $\{s,r\}$ and maintain an optimum solution. However, this replacement decreased the number of cross-links, contradicting our choice of $\textrm{OPT}$.

$$\square $$

Finally, [3] presented a strong rounding technique for points in $x\in P^{\textrm{min}}_{\textrm{bundle}}$, based on a so-called *stack analysis*, which works even for arbitrary (non-Leaf-to-Leaf+ CacAP) instances. The restriction to Leaf-to-Leaf+ instances leads to the result below (Lemma 29). In order to state the rounding result, we need the function $g: [0,1] \rightarrow \mathbb {R}_{\ge 0}$ defined by

$$\begin{aligned} g(\lambda ) :=\lambda \cdot \big (1- e^{-\lambda }\big ) \end{aligned}$$

and the function $\textrm{gain}: [0,1]^2 \rightarrow \mathbb {R}$ defined by

$$\begin{aligned} \textrm{gain}(\lambda , \eta ) :={\left\{ \begin{array}{ll} \lambda \left( e^{- \eta } -1 + \eta \right) \cdot e^{- \lambda + \eta } &{}\text { if }\eta > \frac{1}{2}\lambda \\ \lambda \left( e^{- \eta } -1 + \eta \right) \cdot \left( 1- \lambda + \eta \right) &{} \text { otherwise}; \end{array}\right. } \end{aligned}$$

moreover, for a leaf $t\in T$, we define $\lambda _t^0 :=x(\delta _L(t) \cap L_\mathrm{{cross}})$.

### Lemma 29

([3]) There is a polynomial time algorithm that, given a point $x\in P^{\min }_{\textrm{bundle}}(G,L)$, returns a Leaf-to-Leaf+ CacAP solution $F \subseteq L$ with

9 $$\begin{aligned} |F| \le x(L_{\textrm{in}}) + 2 \cdot x(L_{\textrm{cross}}) - b \cdot \sum _{t\in T} g(\lambda _t^0) \end{aligned}$$

for any $b\in \left[ \frac{5}{12},\frac{1}{2}\right]$ such that for all $v,w \in T$ and for all $s_{vw}$ ($v,w\in T$) and $\eta ^{c_v}_w$ ($v,w\in T$) with $0 \le s_{vw} \le \eta _w^{c_v} \le \lambda _w^0 \le 1$ and $0 \le s_{vw} \le \lambda _v^0 \le 1$, we have

10 $$\begin{aligned} \begin{aligned} 0 \le&\ \frac{b}{\lambda _w^0 - \eta _w^{c_v}} \cdot \textrm{gain}(\lambda _w^0, \eta ^{c_v}_w) \\&\ - s_{vw} \cdot \left( b-\tfrac{1}{3}\right) - (\eta _w^{c_v} - s_{vw}) \cdot \left( 2\left( b-\tfrac{2}{5}\right) -\tfrac{1}{30}\right) \\&+ \max \{0,\ x(S_v) - \eta _w^{c_v}\} \cdot \left( \tfrac{1}{2}-b \right) \\&+ \max \{0,\ 1 - x(S_v) -\eta _w^{c_v} +s_{vw}\} \cdot \left( 1-b\right) . \end{aligned} \end{aligned}$$

Even though Lemma 29 is a direct consequence of the results in [3], we briefly discuss it in Appendix B as it does not appear explicitly in [3].

We now show how we combine the matching-based approach from Sect. 2 with the rounding result given by Lemma 29 to prove Theorem 1.

### Theorem 1

There is a 1.29-approximation algorithm for Leaf-to-Leaf CAP (and therefore also Leaf-to-Leaf TAP).

### Proof

By Theorem 4, it suffices to show that there is a $\rho$-approximation algorithm for *O*(1)-wide instances of Leaf-to-Leaf CacAP for some $\rho < 1.29$.

Let us now describe such a $\rho$-approximation algorithm for *O*(1)-wide instances of Leaf-to-Leaf CacAP. For a *k*-wide instance of Leaf-to-Leaf CacAP, we consider its root-shadow completion (*G*, *L*), which is a Leaf-to-Leaf+ CacAP instance. Let $\textrm{OPT}$ be a weakly $L_\mathrm{{cross}}$-minimal optimum solution of the instance (*G*, *L*), which exists by Lemma 28.

We compute a matching $M\subseteq L$ on the leaves of *G* without bad links that minimizes $|M| + \frac{1}{2}|M_{\textrm{in}}| + (|T|-2|M|)$. By Lemma 11, we have

$$\begin{aligned} |M| + \frac{1}{2}|M_{\textrm{in}}| + (|T|-2|M|) \le |\textrm{OPT}|+\frac{1}{2}|\textrm{OPT}_\mathrm{{in}}|. \end{aligned}$$

Because $\textrm{OPT}$ is weakly $L_\mathrm{{cross}}$-minimal, this implies that the incidence vector $\chi ^{\textrm{OPT}}$ of $\textrm{OPT}$ is a feasible solution to the following linear program:

11 $$\begin{aligned} \begin{array}{crccc} \min &{} x(L) &{} &{} &{} \\ \text {s.t.}&{} |M| + \frac{1}{2}|M_{\textrm{in}}| + (|T|-2|M|) &{} \le &{} x(L)+\frac{1}{2}x(L_{\textrm{in}}) &{} \\ &{} x &{} \in &{} P_{\textrm{bundle}}^{\textrm{min}}(G,L). \end{array} \end{aligned}$$

We compute an optimum solution *x* to the LP (11). Because $\chi ^{\textrm{OPT}}$ is a feasible solution to (11), we have $x(L) \le |\textrm{OPT}|$.

By Lemma 10, we can compute a feasible CacAP solution $F_1$ with

12 $$\begin{aligned} |F_1| \ \le \ |M| + \tfrac{1}{2} |M_\mathrm{{in}}| + (|T| -2|M|)\ \le \ x(L)+\frac{1}{2}x(L_{\textrm{in}}). \end{aligned}$$

Finally, we apply the algorithm from Lemma 29 for $b:=0.452$ to obtain a CacAP solution $F_2$ and return the cheaper of the two solutions $F_1$ and $F_2$.

To verify Condition (10) of Lemma 29 for $b:=0.452$ we use a computer program. Let $\alpha :=\frac{x(L{\textrm{cross}})}{x(L)}$. Then, by (9) from Lemma 29, the CacAP solution $F_2$ fulfills

13 $$\begin{aligned} \begin{aligned} |F_2|\ \le&\ x(L_{\textrm{in}}) + 2 \cdot x(L_{\textrm{cross}}) - b \cdot \sum _{v\in T} g(\lambda ^0_v) \\ =&\ \left( 1 + \alpha - \frac{b}{x(L)} \sum _{v\in T} g(\lambda ^0_v) \right) \cdot x(L) \\ =&\ \left( 1 + \alpha - \frac{b \cdot 2\alpha }{\sum _{v\in T} \lambda ^0_v } \sum _{v\in T} g(\lambda ^0_v) \right) \cdot x(L). \end{aligned} \end{aligned}$$

The solution we return has $\min \{ |F_1|, |F_2|\}$ many links and thus by combining (12), $x(L_{\textrm{in}}) = (1 - \alpha ) \cdot x(L)$, and (13), we obtain an approximation ratio of

$$\begin{aligned} \begin{aligned}&\max \left\{ \ \min \left\{ \ \frac{3}{2}-\frac{1}{2}\alpha , 1 + \alpha - \frac{b \cdot 2 \alpha }{\sum _{v\in T} \lambda ^0_v } \sum _{v\in T} g(\lambda ^0_v)\ \right\} : \right. \\&\qquad \quad \left. \begin{array}{l} \alpha \in [0,1], \ \lambda ^0_v \in [0,1] \ \forall \ v\in T, \\ \alpha \cdot |T|\le \sum _{v\in T} \lambda ^0_v \le |T| \end{array}\right\} . \end{aligned} \end{aligned}$$

Because the function *g* is convex in $\lambda$, replacing $\lambda ^0_v$ by its average value over all of the leaves $v\in T$ does not increase the value of $\sum _{v\in T} g(\lambda ^0_v)$. Moreover, this replacement does not change $\sum _{v\in T} \lambda ^0_v$. Thus, we can simplify the optimization problem and conclude that we obtain an approximation ratio of

$$\begin{aligned} \rho :=\max \Big \{\ \min \Big \{\ \frac{3}{2}-\frac{1}{2}\alpha , 1 + \alpha - \frac{b \cdot 2 \alpha }{\lambda ^0}\cdot g(\lambda ^0)\ \Big \}\, \ 0\le \alpha \le \lambda ^0 \le 1 \Big \}. \end{aligned}$$

As $g(\lambda ) = \lambda \big ( 1-e^{-\lambda }\big )$ and $b=0.452$, this yields

$$\begin{aligned} \rho = \max \Big \{\ \min \Big \{\ \frac{3}{2}-\frac{1}{2}\alpha , 1 + \alpha - 0.904\cdot \alpha \cdot \big ( 1-e^{-\lambda ^0}\big ) \ \Big \}\, \ 0\le \alpha \le \lambda ^0 \le 1 \Big \}. \end{aligned}$$

The optimum value of this optimization problem is attained for $\alpha =\lambda ^0$ being the unique solution to $6\alpha + 9\alpha e^{-\alpha } =5$. This yields $\alpha = \lambda ^0= 0.4202$ and $\rho < 1.29$. $\square$

## A Details of the reduction to $\mathbf {O(1)}$-wide instances from [3]

In this Section we provide details on how Theorem 21 follows from [3]. Theorem 5 in [3] is the analogue of Theorem 21 with $f\equiv 0$. The same proof can be extended to the case of a general polynomial-time computable function *f*. We will first provide a brief outline of the overall proof and then provide adapted versions of those lemmas and proofs from [3] that require (small) changes in order to obtain Theorem 21.

Given an instance $\mathcal {I}=(G,L)$, let $P_{\textrm{CacAP}}(\mathcal {I})$ be the convex hull of all actual solutions, namely

$$\begin{aligned} P_{\textrm{CacAP}}(\mathcal {I}) :=\text {conv}(\{\chi ^F : F\subseteq L, (V,E\cup F) \text { is }3\text { -edge-connected}\})\ . \end{aligned}$$

The proof of Theorem 21 is based on the round-or-cut framework. We will show that there exists a polynomial-time algorithm that, given a point $x\in [0,1]^L$, returns either

(i) a CacAP solution with at most
  $$\begin{aligned} \alpha \cdot (1+\varepsilon )\cdot \textrm{OPT}(\mathcal {I})\quad +\ \max _{\begin{array}{c} \mathcal {S}\!\text { an }(\varepsilon \cdot x(L),k)-\\ \text {splitting of }\mathcal {I} \end{array}} \quad \sum _{\mathcal {J}\!\text { split minor of }\mathcal {S}} f(\mathcal {J}) \end{aligned}$$
  many links, or
(ii) a vector $w\in \mathbb {R}^L$ such that $w^T x < w^T x'$ for all $x' \in P_{\textrm{CacAP}}(G,L)$.

Having such an algorithm, we guess the number $|\textrm{OPT}(\mathcal {I})|$ of links in an optimum solution and run the Ellipsoid method to find a point in the polytope $\{x \in P_{\textrm{CacAP}}(\mathcal {I}): x(L) = |\textrm{OPT}(\mathcal {I})| \}$. As a separation oracle, we can return a separating hyperplane if the given point $x\in [0,1]^L$ does not fulfill $x(L)= |\textrm{OPT}(\mathcal {I})|$ and otherwise apply the algorithm that returns either a CacAP solution as in (i) or a separating hyperplane as in (ii). If we obtain a CacAP solution as in (i), this solution has the desired properties (as required in Theorem 21) because $x(L)= |\textrm{OPT}(\mathcal {I})|$; in this case we are done and we terminate the Ellipsoid method. Otherwise, we have obtained the separating hyperplane that we need to continue with the Ellipsoid method. Because the Ellipsoid terminates in polynomial time, it indeed suffices to provide an algorithm that given a point $x\in [0,1]^L$ either returns (i) or (ii). Polynomial-time termination of the Ellipsoid Method follows by classical results (see [17, Theorem (6.4.1)]) because the polytope $\{x \in P_{\textrm{CacAP}}(\mathcal {I}): x(L) = |\textrm{OPT}(\mathcal {I})| \}$, over which we run the Ellipsoid Method, has facet complexity that is bounded polynomially in |*L*|, because it is a $\{0,1\}$-polytope in $[0,1]^L$.

We now give a brief outline of the algorithm used to round $x\in [0,1]^L$ or separate *x* from $P_{\textrm{CacAP}}(G,L)$. The algorithm proceeds in three steps:

- The first step, called *heavy cut covering*, computes a set $L_H \subseteq L$ of links with $|L_H|\le \frac{\varepsilon }{2}x(L)$ that covers all *x*-*heavy cuts*, i.e., we have $L_H \cap \delta _L(C) \ne \emptyset$ for all 2-cuts $C\in \mathcal {C}$ with $x(\delta _L(C)) > \tfrac{16}{\varepsilon }$. Then we consider the residual instance with respect to $L_H$. [3, Lemma 22] shows that this residual instance has no heavy cuts. Moreover, if we take any solution *F* for the residual instance, then $F\cup L_H$ is a feasible solution for $\mathcal {I}$ (by [3, Corollary 20]).
- In the second step, we split the instance into *k*-wide instances using the splitting operations from Definition 17.
- Finally, for every split minor arising from the splitting procedure we either find a CacAP solution or we obtain a separating hyperplane. If we find a CacAP solution for all of the split minors, we merge them to a CacAP solution of the overall instance.

Let us now recap the main statement from [3] that we use for merging the CacAP solutions for the split minors to a solution of the overall instance. Recall that splitting an instance $\mathcal {I}$ at a cut $C\in \mathcal {C}$ leads to two sub-instances $\mathcal {I}_C$ and $\mathcal {I}_{V\setminus C}$, where $\mathcal {I}_C$ is the instance obtained from $\mathcal {I}$ by contracting all vertices except for those in *C*, and $\mathcal {I}_{V\setminus C}$ is the instance obtained from $\mathcal {I}$ by contracting *C*. If we want to merge solutions $F_{C}$, $F_{V\setminus C} \subseteq L$ to the sub-instances $\mathcal {I}_C$ and $\mathcal {I}_{V{\setminus } C}$, respectively, to a solution of $\mathcal {I}$, it may be not enough to simply take the union of $F_C$ and $F_{V{\setminus } C}$. However, we can bound the number of links that need to be added to $F_C\cup F_{V{\setminus } C}$ to obtain a solution to $\mathcal {I}$ as shown in [3].

### Proposition 30

(Proposition 11, [3]) Given a feasible CacAP instance $\mathcal {I}=(G=(V,E),L)$, a 2-cut $C\in \mathcal {C}$, and solutions $F_C, F_{V{\setminus } C} \subseteq L$ to $\mathcal {I}_C$ and $\mathcal {I}_{V{\setminus } C}$, respectively, one can compute a link set $F\subseteq L$ in polynomial time such that

(i) $F_C \cup F_{V\setminus C} \cup F$ is a CacAP solution to $\mathcal {I}$, and
(ii) $$|F|\le |\delta _L(C)\cap F_C|-1$$.

We now explain in detail those parts of the proof from [3] that need to be adapted to obtain Theorem 21. First, we provide a generalization of Lemma 32 from [3], which yields an algorithm for *k*-wide instances that either returns a cheaply mergeable solution or a separating hyperplane. In this algorithm we will use the fact that the function *f* is polynomial-time computable. We need the following definition.

### Definition 31

($\lambda$-contraction minor) For an instance $\mathcal {I}=(G,L)$, a $\lambda$-*contraction minor* is an instance $\mathcal {I}'$ that can be obtained by $\mathcal {I}$ by

- deleting links, and
- applying contraction operations (see Definition 18) for at most $\lambda$ many links.

Now we can generalize Lemma 32 from [3] as follows.^3^

### Lemma 32

Let $\varepsilon \ge 0$ and $\varepsilon ' = \frac{\varepsilon }{4}$. Suppose there is an polynomial-time algorithm $\mathcal {A}$ that for any *k*-wide CacAP instance $\mathcal {J}$ returns a solution of cost at most $\alpha \cdot \textrm{OPT}(\mathcal {J}) + f(\mathcal {J})$, where *f* is a function that is polynomial-time computable. Then there is a polynomial-time algorithm that, given a *k*-wide CacAP instance $\mathcal {I}=(G=(V,E),L)$, a vector $x\in [0,1]^L$, and a vertex *s* of *G* with $|\delta _E(s)|=2$ and $x(\delta _L(s))\le \frac{16}{\varepsilon }$, either returns

- a CacAP solution $F\subseteq L$ with
  $$\begin{aligned} \begin{aligned} |F| + |\delta _{F}(s)| \le \&(1+\varepsilon ')\cdot \alpha \cdot \left( x(L) + x(\delta _L(s))\right) \\&+ \max \{f(\mathcal {I}') :\mathcal {I}' \text { is a }\lambda -\text { contraction minor of }\mathcal {I}\}, \end{aligned} \end{aligned}$$
  where $$\lambda = \frac{1+\varepsilon '}{\varepsilon '}\cdot \frac{16}{\varepsilon }$$, or
- a vector $w\in \mathbb {R}^L$ such that $w^T x < w^T x'$ for all $x' \in P_{\textrm{CacAP}}(\mathcal {I})$.

### Proof

The proof of this lemma follows the proof of Lemma 32 in [3]. Let $r\in V$ be a *k*-wide root. If $x(\delta _L(s))<1$, we have $x(\delta _L(s)) < x'(\delta _L(s))$ for all $x'\in P_{\textrm{CacAP}}(\mathcal {I})$ and can thus return $w=\chi ^{\delta _L(s)}$. Otherwise, we proceed as follows. For every set $S \subseteq \delta _L(s)$ with $|S| \le \lambda$, we apply the given algorithm $\mathcal {A}$ for *k*-wide instances to the residual instance $\mathcal {I}'$ of $(G, L\setminus \delta _L(s))$ with respect to *S*. This instance $\mathcal {I}'$ arises from (*G*, *L*) by applying contraction operations for the links in *S* after deleting all links in $\delta _L(s)$. Thus, $\mathcal {I}'$ is a $\lambda$-contraction minor and it is *k*-wide by [3, Lemma 31].

If for some such set *S*, the algorithm $\mathcal {A}$ returns a solution $F'$ with $|F'| + 2 |S| \le (1+\varepsilon ')\cdot \alpha \cdot \left( x(L) + x(\delta _L(s))\right) + f(\mathcal {I}')$, we return $F=F' \cup S$. Here we use the fact that *f* is a polynomial-time computable function; hence, we can check in polynomial time whether we are in this case. Otherwise, we define $\mu := 1 + \frac{\varepsilon ' ( x(L) + x(\delta _L(s)) ) }{ x(\delta _L(s)) }$ and claim that

14 $$\begin{aligned} x'(L)+ \mu \cdot x'(\delta _L(s)) > x(L) + \mu \cdot x(\delta _L(s)) \quad \forall \; x'\in P_{\textrm{CacAP}}(\mathcal {I}), \end{aligned}$$

which leads to a vector $w\in \mathbb {R}^L$ as desired. Suppose that (14) does not hold. Then there exists a solution $F^*$ of $\mathcal {I}$ with

15 $$\begin{aligned} |F^*| + \mu \cdot | \delta _{F^*}(s) | \le x(L) + \mu \cdot x(\delta _L(s)) = (1+\varepsilon ') \cdot (x(L) + x(\delta _L(s)). \end{aligned}$$

For $S = \delta _{F^*}(s)$ this implies

$$\begin{aligned} \frac{\varepsilon ' \left( x(L) + x(\delta _L(s)) \right) }{ x(\delta _L(s)) } \cdot |S|\ \le \ \mu \cdot |S|\ \le \ (1+\varepsilon ') \cdot \left( x(L) + x(\delta _L(s)) \right) \end{aligned}$$

and hence

$$\begin{aligned} |S| \ \le \ \frac{1+\varepsilon '}{\varepsilon '} x(\delta _L(s))\ \le \ \lambda . \end{aligned}$$

Thus, we considered the set *S* in our algorithm described above. Let $F'$ be the output of the $\alpha$-approximation algorithm $\mathcal {A}$ applied to the residual instance $\mathcal {I}'$ of $(G, L{\setminus } \delta _L(s))$ with respect to *S*. Then $|F'| \le \alpha \cdot |F^* {\setminus } S|+f(\mathcal {I}')$ because the set $F^*{\setminus } S$ is a feasible solution of this residual instance (see [3, Corollary 20]). Therefore, using $\alpha \ge 1$, $S=\delta _{F^*}(s)$, and (15), we get

$$\begin{aligned} \begin{aligned} |F' | + 2 |S|&\le \alpha \cdot |F^*\setminus S|+f(\mathcal {I}') + 2 |S| \\ {}&\le \alpha \cdot (|F^*| + |S|)+f(\mathcal {I}') \\ {}&\le \alpha \cdot (|F^*| + \mu |\delta _{F^*}(s)|) + f(\mathcal {I}')\\&\le (1+\varepsilon ')\cdot \alpha \cdot (x(L) + x(\delta _L(s))+f(\mathcal {I}'), \end{aligned} \end{aligned}$$

contradicting the fact that we did not return $F' \cup S$.

$$\square $$

The next lemma provides a generalization of Lemma 33 from [3] (except that we choose *k* slightly differently). This lemma extends the algorithm that, given $x\in [0,1]^L$, either rounds it to a cheap solution or provides a separating hyperplane from *k*-wide instances to general instances without *x*-heavy cuts.

### Lemma 33

Let $0 \le \varepsilon \le 1$ and $k:=\frac{64(8+3\varepsilon )}{\varepsilon ^3}$. Suppose there exist a polynomial-time algorithm $\mathcal {A}$ that for any *k*-wide CacAP instance $\mathcal {J}$ returns a solution of cost at most $\alpha \cdot \textrm{OPT}(\mathcal {J}) + f(\mathcal {J})$, where *f* is a function that is computable in polynomial time. Then, for any CacAP instance $\mathcal {I}=(G=(V,E),L)$ and $x\in [0,1]^L$ such that no cut is *x*-heavy, there is a polynomial-time algorithm that computes either

- a CacAP solution $L'$ with
  $$\begin{aligned} |L'|\le \alpha \cdot \left( 1+\frac{\varepsilon }{2}\right) \cdot x(L) +\ \max _{\begin{array}{c} \mathcal {S}\!\text { a }(\gamma ,k)-\\ \text {splitting of }\mathcal {I} \end{array}}\quad \sum _{\mathcal {J}\!\text { split minor of }\mathcal {S}} f(\mathcal {J}), \end{aligned}$$
  where $\gamma =\frac{\varepsilon }{4}\cdot |T|$ with *T* being the set of leaves of *G*, or
- a vector $w\in \mathbb {R}^L$ such that $w^T x < w^T x'$ for all $x' \in P_{\textrm{CacAP}}(\mathcal {I})$.

### Proof

The proof of this lemma follows from Lemma 33 in [3] and proceeds by induction on the number of vertices. We fix an arbitrary root $r\in V$. If there is no 2-cut $C\subsetneq V{\setminus } \{r\}$ such that $|C\cap T|> \tfrac{k}{2}$, then, following the terminology of [3], the instances is called *unsplittable*. (More precisely, for an instance to be unsplittable no such cut *C* must exist that is not *x*-heavy; however, as no *x*-heavy cuts exist in our instance by assumption, this condition can be dropped here.) By [3, Lemma 12], every unsplittable instance is *k*-wide, and we can thus apply algorithm $\mathcal {A}$ to $\mathcal {I}$ to obtain a feasible solution $L'$. If $|L'| \le \alpha \cdot x(L) + f(\mathcal {I})$, then

$$\begin{aligned} |L'| \le \alpha \cdot x(L) + f(\mathcal {I}) \le \alpha \cdot \left( 1+\frac{\varepsilon }{2}\right) \cdot x(L) +\ \max _{\begin{array}{c} \mathcal {S}\!\text { a }(\gamma ,k)-\\ \text {splitting of }\mathcal {I} \end{array}}\quad \sum _{\mathcal {J}\!\text { split minor of }\mathcal {S}} f(\mathcal {J}), \end{aligned}$$

because $\mathcal {I}$ itself is a $(\gamma ,k)$-split-minor. In this case we return $L'$. Otherwise, we return $w = \chi ^{L}$, which fulfills $w^T x < w^T x'$ for all $x' \in P_{\textrm{CacAP}}(\mathcal {I})$ because $\mathcal {A}$ returns a solution of cost at most $\alpha \cdot \textrm{OPT}(\mathcal {J}) + f(\mathcal {J})$.

Hence, we may assume that $\mathcal {I}$ is splittable at some cut *C*, i.e., there is a cut *C* that fulfills $|T\cap C| > \tfrac{k}{2}$ and $C\subsetneq V\setminus \{r\}$. We choose $C\in \mathcal {C}$ to be a minimal 2-cut with these properties.

We denote by $\mathcal {I}_C=(G_C, L_C)$ the instance arising from $\mathcal {I}$ by contracting $V\setminus C$ and we choose as new root the vertex *s* arising from the contraction of $V\setminus C$. (Note that $\{r\} \subsetneq V\setminus C$.) Because *C* was chosen minimally, the instance $\mathcal {I}_C$ with root *s* is *k*-wide (this is again a consequence of [3, Lemma 12]). We apply Lemma 32 to $\mathcal {I}_C$ to either obtain a CacAP solution $F_C$ for $\mathcal {I}_C$ with

16 $$\begin{aligned} \begin{aligned} |F_C| + |\delta _{F_C}(s)| \le&\left( 1+\tfrac{\varepsilon }{4}\right) \cdot \alpha \cdot (x(L_C) + x(\delta _L(s))) \\&+ \max \{f(\mathcal {I}') :\mathcal {I}' \text { is a } \lambda -\text { contraction minor for }\mathcal {I}_C\}, \end{aligned} \end{aligned}$$

where $\lambda = \frac{1+\varepsilon '}{\varepsilon '}\cdot \frac{16}{\varepsilon }$, or a vector $w_C\in \mathbb {R}^{L_C}$ satisfying $w_{C}^T x_{C} < w_{C}^T x'$ for all $x' \in P_{\textrm{CacAP}}(\mathcal {I}_C)$, where $x_{C}$ is the restriction of the vector *x* to the links in $L_C$.

Moreover, we denote by $\mathcal {I}_{V{\setminus } C}=(G_{V{\setminus } C}, L_{V{\setminus } C})$ the CacAP instance arising from $\mathcal {I}$ by contracting *C*. By induction, we can obtain a CacAP solution $F_{V\setminus C}$ for $\mathcal {I}_{V\setminus C}$ such that

17 $$\begin{aligned} |F_{V\setminus C}|\le \alpha \cdot \left( 1+\tfrac{\varepsilon }{2}\right) \cdot x(L_{V\setminus C}) + \max _{\begin{array}{c} \mathcal {S}\!\text { a }(\gamma ',k)-\\ \text {splitting of }\mathcal {I}_{V\setminus C} \end{array}}\quad \sum _{\mathcal {J}\!\text { split minor of }\mathcal {S}} f(\mathcal {J}), \end{aligned}$$

where $\gamma '= \tfrac{\varepsilon }{4} \cdot |T_{V\setminus C}|$ with $T_{V\setminus C}$ being the set of leaves of $G_{V\setminus C}$, or a vector $w_{\bar{C}}\in \mathbb {R}^{L_{V\setminus C}}$ such that $w_{\bar{C}}^T x_{\bar{C}} < w_{\bar{C}}^T x'$ for all $x' \in P_{\textrm{CacAP}}(\mathcal {I}_{V{\setminus } C})$, where $x_{\bar{C}}$ is the vector *x* restricted to the entries corresponding to links in $L_{V\setminus C}$.

If we obtained a vector $w_C\in \mathbb {R}^{L_C}$ such that $w_C^T x_C < w_C^T x'$ for all $x' \in P_{\textrm{CacAP}}(\mathcal {I}_C)$, we can extend it to a vector $w\in \mathbb {R}^L$ such that $w^T x < w^T x'$ for all $x' \in P_{\textrm{CacAP}}(\mathcal {I})$ by setting $w_\ell =0$ for all $\ell \in L {\setminus } L_C$. Here we use that restricting a CacAP solution *F* of $\mathcal {I}$ to $F\cap L_C$ yields a CacAP solution for $\mathcal {I}_C$. We can proceed analogously if we have a vector $w_{\bar{C}}\in \mathbb {R}^{L_{V{\setminus } C}}$ such that $w_{\bar{C}}^T x < w_{\bar{C}}^T x'$ for all $x' \in P_{\textrm{CacAP}}(\mathcal {I}_{V{\setminus } C})$.

Otherwise, we obtained solutions $F_C$ and $F_{V{\setminus } C}$ as discussed above and apply Proposition 30. This yields a set $F\subseteq L$ such that $F \cup F_C \cup F_{V{\setminus } C}$ is a CacAP solution for $\mathcal {I}$ and $|F| \le |F_C \cap \delta _L(C)| = |\delta _{F_C}(s)|$. Thus, combining (16) and (17), we obtain

18 $$\begin{aligned} |F \cup F_C \cup F_{V\setminus C}|\ \le&\ |F_{V\setminus C}| + |F_C| + |\delta _{F_C}(s)| \nonumber \\ \le&\ \alpha \cdot \left( 1+\tfrac{\varepsilon }{2}\right) \cdot x(L_{V\setminus C}) + \max _{\begin{array}{c} \mathcal {S}\!\text { a }(\gamma ',k)-\\ \text {splitting of }\mathcal {I}_{V\setminus C} \end{array}}\quad \sum _{\mathcal {J}\!\text { split minor of }\mathcal {S}} f(\mathcal {J}) \nonumber \\&\ + \left( 1+\tfrac{\varepsilon }{4}\right) \cdot \alpha \cdot (x(L_C) + x(\delta _L(s))) \nonumber \\ {}&\ + \max \{f(\mathcal {I}') :\mathcal {I}' \text { is a }\lambda -\text { contraction minor for }\mathcal {I}_C\} \nonumber \\ =&\ \alpha \left( 1+\tfrac{\varepsilon }{2}\right) x(L_{V\setminus C}) +\alpha \left( 1+\tfrac{\varepsilon }{2}\right) x(L_C) - \alpha \tfrac{\varepsilon }{4} \cdot x(L_C) \nonumber \\&\ + \alpha \left( 1+\tfrac{\varepsilon }{4}\right) \cdot x(\delta _L(C)) \nonumber \\&\ + \max _{\begin{array}{c} \mathcal {S}\!\text { a }(\gamma ',k)-\nonumber \\ \text {splitting of }\mathcal {I}_{V\setminus C} \end{array}}\quad \sum _{\mathcal {J}\!\text { split minor of }\mathcal {S}} f(\mathcal {J}) \nonumber \\ {}&\ + \max \{f(\mathcal {I}') :\mathcal {I}' \text { is a }\lambda -\text { contraction minor for }\mathcal {I}_C\} \nonumber \\ =&\ \alpha \left( 1+\tfrac{\varepsilon }{2}\right) \cdot x(L) + \alpha \left( 2+\tfrac{3\varepsilon }{4}\right) \cdot x(\delta _L(C)) - \alpha \tfrac{\varepsilon }{4} \cdot x(L_C) \nonumber \\&\ + \max _{\begin{array}{c} \mathcal {S}\!\text { a }(\gamma ',k)-\nonumber \\ \text {splitting of }\mathcal {I}_{V\setminus C} \end{array}}\quad \sum _{\mathcal {J}\!\text { split minor of }\mathcal {S}} f(\mathcal {J}) \nonumber \\ {}&\ + \max \{f(\mathcal {I}') :\mathcal {I}' \text { is a }\lambda -\text { contraction minor for }\mathcal {I}_C\}, \end{aligned}$$

where we used $L_C \cup L_{V{\setminus } C} = L$ and $L_C \cap L_{V{\setminus } C} = \delta _L(C)$. Because $|C\cap T| > \tfrac{k}{2}$, we have $x(L_C) \ge \frac{k}{4} = 16\cdot \frac{8+3\varepsilon }{\varepsilon ^3}$. Moreover, $x(\delta _L(C))\le \frac{16}{\varepsilon }$ because $\delta _L(C)$ is not *x*-heavy. This implies

$$\begin{aligned} \left( 2+\tfrac{3\varepsilon }{4}\right) \cdot x(\delta _L(C))\ \le \ \left( 2+\tfrac{3\varepsilon }{4}\right) \cdot \tfrac{16}{\varepsilon }\ \le \ \tfrac{\varepsilon }{4} \cdot 16\cdot \tfrac{8+3\varepsilon }{\varepsilon ^3}\ \le \ \tfrac{\varepsilon }{4}\cdot x(L_C). \end{aligned}$$

Together with (18), we obtain

19 $$\begin{aligned} \begin{aligned} |F \cup F_C \cup F_{V\setminus C}|\ \le&\ \alpha \cdot \left( 1+ \tfrac{\varepsilon }{2}\right) \cdot x(L)\\&\ + \max \{f(\mathcal {I}') :\mathcal {I}' \text { is a }\lambda -\text { contraction minor for }\mathcal {I}_C\} \\&\ + \max _{\begin{array}{c} \mathcal {S}\!\text { a }(\gamma ',k)-\\ \text {splitting of }\mathcal {I}_{V\setminus C} \end{array}}\quad \sum _{\mathcal {J}\!\text { split minor of }\mathcal {S}} f(\mathcal {J}). \end{aligned} \end{aligned}$$

Let $\mathcal {I}'_C \in \text {argmax}\{f(\mathcal {I}') :\mathcal {I}' \text { is a } \lambda -\text { contraction minor for }\mathcal {I}_C\}$. It remains to prove the following claim.

### Claim

$$\begin{aligned} f(\mathcal {I}'_C) + \max _{\begin{array}{c} \mathcal {S}\!\text { a } (\gamma ',k)-\\ \text {splitting of }\mathcal {I}_{V\setminus C} \end{array}}\quad \sum _{\mathcal {J}\!\text { split minor of }\mathcal {S}} f(\mathcal {J}) \le \max _{\begin{array}{c} \mathcal {S}\!\text { a } (\gamma ,k)-\\ \text {splitting of }\mathcal {I} \end{array}}\quad \sum _{\mathcal {J}\!\text { split minor of }\mathcal {S}} f(\mathcal {J}). \end{aligned}$$

### Proof

Let $\mathcal {J}_1,\dots ,\mathcal {J}_N$ be the split minors of a $(\gamma ',k)$-splitting of $\mathcal {I}_{V\setminus C}$ that maximize

$$\begin{aligned} \max _{\begin{array}{c} \mathcal {S}\!\text { a }(\gamma ',k)-\\ \text {splitting of }\mathcal {I}_{V\setminus C} \end{array}} \quad \sum _{\mathcal {J}\!\text { split minor of }\mathcal {S}} f(\mathcal {J}). \end{aligned}$$

We finish the proof by showing that $\mathcal {I}'_C,\mathcal {J}_1,\dots ,\mathcal {J}_N$ are the split minors of a $(\gamma ,k)$-splitting of $\mathcal {I}$. By construction, all $\mathcal {I}'_C,\mathcal {J}_1,\dots ,\mathcal {J}_N$ are obtained after applying a sequence of splitting and contraction operations and deletions of links. Moreover, they are all *k*-wide. We only need to prove that the number of contractions is at most $\gamma = \tfrac{\varepsilon }{4}\cdot |T|$.

Recall we choose the cut *C* to split at such that $|C\cap T| > \tfrac{k}{2}$, implying $|T_{V{\setminus } C}| \le |T| - \tfrac{k}{2} + 1$. Therefore, because $\mathcal {J}_1,\dots ,\mathcal {J}_N$ are the split minors of a $(\gamma ',k)$-splitting of $\mathcal {I}_{V\setminus C}$, the number of contractions applied to obtain $\mathcal {J}_1,\dots ,\mathcal {J}_N$ from $\mathcal {I}_{V\setminus C}$ (by splitting, contraction, and deletion of links) is at most $\gamma ' = \tfrac{\varepsilon }{4} \cdot |T_{V{\setminus } C}| \le \tfrac{\varepsilon }{4} \cdot (|T| - \tfrac{k}{2} + 1)$. The total number of contractions to obtain $\mathcal {I}'_C,\mathcal {J}_1,\dots ,\mathcal {J}_N$ is thus at most

$$\begin{aligned} \lambda + \tfrac{\varepsilon }{4} \cdot (|T| - \tfrac{k}{2} + 1) \le \tfrac{\varepsilon }{4} \cdot |T| = \gamma \end{aligned}$$

because

$$\begin{aligned} \lambda +\frac{\varepsilon }{4}= \frac{1+\varepsilon '}{\varepsilon '}\cdot \frac{16}{\varepsilon }+\frac{\varepsilon }{4} =\frac{1+\varepsilon /4}{\varepsilon /4}\cdot \frac{16}{\varepsilon }+\frac{\varepsilon }{4} \le \frac{\varepsilon }{8}\cdot \frac{64(8+3\varepsilon )}{\varepsilon ^3}= \frac{\varepsilon }{4}\cdot \frac{k}{2}. \end{aligned}$$

$$\square $$ $$\square $$

In order to obtain an algorithm that, given a point $x\in [0,1]^L$, returns either a CacAP solution as in (i) or a vector $w\in \mathbb {R}^L$ as in (ii), we proceed as follows. First, we observe that if $x(L) < |T|/2$ we can return a vector *w* as in (ii) because $x'(L) \ge |T|/2$ for every vector $x'\in P_{\textrm{CacAP}}(\mathcal {I})$. Hence, we will assume in the following that this is not the case.

Given $x\in [0,1]^L$ and $\mathcal {W}= \{C\in \mathcal {C}: C\text { is }x-\text { heavy}\}$, we apply [3, Theorem 23] to obtain a cheap heavy cut covering, i.e., a set $L_H\subseteq L$ of links covering all heavy cuts with $|L_H|\le \frac{\varepsilon }{2}\cdot x(L)$. Then we consider the residual instance $\mathcal {I}_H$ of $\mathcal {I}$ with respect to $L_H$ (see Definition 12) and apply Lemma 33. Because $\mathcal {I}_H$ arises from $\mathcal {I}$ by the contraction of $|L_H|$ many links, every $(\gamma , k)$-splitting of $\mathcal {I}_H$ is a $(\gamma +|L_H|, k)$-splitting of $\mathcal {I}$. Hence, the application of Lemma 33 yields either a separating hyperplane or a CacAP solution *F* for $\mathcal {I}_H$ with

$$\begin{aligned} |F|\le \alpha \cdot \left( 1+\frac{\varepsilon }{2}\right) \cdot x(L) +\ \max _{\begin{array}{c} \mathcal {S}\!\text { an }(\varepsilon \cdot x(L),k)-\\ \text {splitting of }\mathcal {I} \end{array}}\quad \sum _{\mathcal {J}\!\text { split minor of }\mathcal {S}} f(\mathcal {J}), \end{aligned}$$

where we used $\gamma + |L_H| \le \tfrac{\varepsilon }{4} \cdot |T| + \frac{\varepsilon }{2}\cdot x(L) \le \varepsilon \cdot x(L)$. Then [3, Corollary 20] implies that $F\cup L_H$ is a solution for the instance $\mathcal {I}$ with the desired guarantee (i).

## B Deriving Lemma 29 from [3]

Lemma 29 is a consequence of the stack analysis presented in [3], which was performed for general CAP instances. To obtain Lemma 29 for the leaf-to-leaf case, we can use exactly the same analysis with the following minor differences:

- In [3] there is a distinction between different types of cross links (see Section 6.4 [3]). In leaf-to-leaf instances only cross links of a single type exist, which implies that the values $\lambda _t^1$, $\mu ^0_t$, and $\mu ^1_t$ appearing in [3] are all equal to zero and thus we can eliminate their occurrences. In particular, our definitions of the functions *g* and $\textrm{gain}$ then coincide with those from [3]. Moreover, Condition (10) is for leaf-to-leaf+ instances equivalent to equation (28) in [3].
- Formally, [3] considered general *k*-wide instances of CacAP and defined $P^{\textrm{min}}_{\textrm{bundle}}(G,L)$ in a slightly different way, requiring that the vectors $x|_{L_i}$ for $i\in \{1,\dots ,p\}$ are convex combinations of incidence vectors of $L_{\textrm{cross}}$-minimal solutions (instead of weakly $L_{\textrm{cross}}$-minimal solutions). However, this stronger property is used only in a single place of the proof, namely the proof of Lemma 53 in [3], which is a trivial statement for leaf-to-leaf+ instances. This is because Lemma 53 from [3] provides a lower bound on $x(L_{\textrm{up}})$ where $L_{\textrm{up}} \subseteq L$. This lower bound is 0 for every leaf-to-leaf+ instance and thus the statement of Lemma 53 from [3] trivially holds in our setting.
- In [3] the value *b* is fixed to 0.42 which leads to the best approximation guarantee for general CAP instances. However, the only two conditions on *b* that are used in the proof are equation (28) in [3], which is equivalent to Condition (10) stated in Lemma 29, and $b\in [\frac{5}{12},\frac{1}{2}]$. (The latter condition is equivalent to the condition $z_1\le z_2 \le 0$ below equation (18) in [3].)

Then Lemma 29 follows from Lemma 59 in [3].

## Footnotes

1 Consider a general weighted TAP instance $G=(V,E)$ with links  and weights $w:L\rightarrow \mathbb {R}_{\ge 0}$. To transform it into a leaf-to-leaf instance, one can do the following operation for each vertex *v*: (i) Add two new vertices $w^1_v, w^2_v$ to the graph and connect each of them only to *v*; hence, $w^1_v$ and $w^2_v$ are leaves; (ii) For each link $\ell \in L$ that has *v* as an endpoint, replace the endpoint *v* by $w^1_v$; (iii) Add a new link $\{w^1_v,w^2_v\}$ of cost 0. One can easily observe that the original instance is equivalent to the obtained leaf-to-leaf one, because, after including the newly added 0-cost links, one falls back to the original instance. An analogous construction works to reduce weighted CAP to weighted Leaf-to-Leaf CAP.

2 We highlight that, in the context of TAP, (dir-LP) is equivalent to the well-known integral up-link LP, which first replaces each link $\{u,v\}$ by at most two other links, one from each endpoint of the link to the vertex of the unique *u*-*v* path in the tree that lies closest to the root.
